# Peer victimization disparities for sexual and gender diverse youth: Cross‐informant reports and associated mental health

**DOI:** 10.1111/jora.70283

**Published:** 2026-10-06

**Authors:** Jingyi Huang, Tessa M. L. Kaufman, Susan Branje

**Affiliations:** ^1^ Department of Youth and Family, Faculty of Social and Behavioural Sciences Utrecht University Utrecht The Netherlands

**Keywords:** mental health, multi‐informants, peer victimization, sexual and gender diversity

## Abstract

Sexual and gender diverse (SGD) youth experience poorer well‐being than their heterosexual, cisgender (HC) peers, partly due to elevated peer victimization. This study examined how youth's and adults' (i.e., parents' and teachers') recognition of peer victimization is related to SGD youth's mental health discrepancies. Using data from the British Millennium Cohort Study, we analyzed congruence in peer victimization reports from parent–youth dyads (Waves 4–6, *n* = 8260–8846) and teacher–youth dyads (Waves 4–5, *n* = 5354–5708). Youth were categorized into four cross‐informant victimization classes: non‐victims, only‐youth‐perceived victims, only‐adult‐perceived victims, and congruent victims. Results showed that parents and teachers generally underestimated peer victimization, with most incidents recognized only by youth themselves. Nevertheless, SGD youth were more likely than HC youth to be classified as congruent victims, indicating their victimization was more visible to parents and teachers. Congruent victims reported the worst mental health, and this cross‐informant recognition pattern was most consistently linked to SGD youth's mental health discrepancies, both concurrently and longitudinally. These findings suggest that while parents and teachers are more aware of SGD youth's victimization than of HC youth's victimization, this awareness has not translated into effective support. This visibility represents a critical intervention opportunity for parents and teachers to provide timely responses that could improve SGD youth's mental health.

## INTRODUCTION

In recent decades, the acceptance of sexual and gender diversity has increased globally, with mostly Western countries, including the United Kingdom, leading the trend (Flores, 2019; ILGA‐Europe, 2026; Wilson, 2020). However, substantial mental health disparities persist between sexual and gender diverse (SGD) youth and their heterosexual, cisgender (HC) peers in the United Kingdom and worldwide, including higher rates of depressive symptoms, self‐harm, substance use, and suicide attempts (Amos et al., 2020; White et al., 2023; Whittle et al., 2024). These health disparities are closely linked to SGD youth's victimization experiences (Russell & Fish, 2016, 2019), as nearly half of British SGD youth experience victimization in schools (Stonewall, 2017). Although adolescence is a milestone period for SGD youth (Baams & Kaufman, 2023), psychosocial vulnerabilities that contribute to their risk of victimization can emerge in childhood (Huang et al., 2024). Therefore, it is crucial to investigate experiences of SGD youth in childhood, even before they are able to feel comfortable or be permitted by adults to report their SGD identities. Youth who later identify as SGD might recognize themselves as being different from the majority social norm (Mustanski et al., 2014) and feel misrepresented and marginalized (Suen et al., 2020), which taxes their mental and interpersonal development. Indeed, studies that linked SGD status reported at later ages back to youth's early development revealed that victimization disparities for SGD youth emerge already in childhood and are explained by a series of psychological and relational challenges (Huang et al., 2024; Martin‐Storey & Fish, 2019).

Parents and teachers are important adult figures in youth development and play a crucial role in recognizing and responding to youth's peer victimization; yet their perceptions may not align with youth's. Examining parent–youth‐ and teacher–youth‐reported victimization can offer valuable insight into youth mental health. For instance, victimization recognized by both youth and parents (congruent reports) or by only one informant (incongruent reports) has been linked to worse psychosocial adjustment, though evidence is mixed regarding which recognition pattern is most detrimental (Goodman, 2013; Tremblay‐Perreault et al., 2022). This highlights the need to examine not only whether victimization occurs but also who recognizes it. Research indicated that parents and teachers can underestimate victimization among SGD youth or report different developmental tendencies than youth themselves (Kaufman et al., 2020; Martin‐Storey & Fish, 2019). However, it is unclear whether (in)congruence in victimization reports differs between SGD and HC youth and how these patterns relate to mental health. This study, therefore, aimed to examine cross‐informant victimization patterns across British SGD and HC youth and their parents and teachers and test how disparities in victimization patterns were associated with youth's mental health outcomes.

### Cross‐informant victimization patterns among SGD youth

Victimization can be difficult to detect, even by close others such as parents or teachers. Yet, beyond self‐report, information from parents and teachers is an essential indicator of youth's victimization and correlated adjustment (Kretschmer et al., 2025). Parents and teachers are likely to underestimate peer victimization experienced by youth (Volk et al., 2017), with teachers underestimating more than parents (Demaray et al., 2013). This might reflect that parents and teachers observe youth's experiences only under specific, and potentially different, circumstances. Parents have less chance to directly observe peer victimization and rather depend on youth's disclosure (Kretschmer et al., 2025). Similar to parents, teachers also learn about victimization through students' disclosure. Differently, teachers have a greater chance to observe overt victimization and group dynamics in the school context (Volk et al., 2017). In other cases, parents and teachers can recognize meaningful victimization incidents when youth do not. For instance, some victimized youth may normalize or minimize such experiences, while others may not realize that those hurtful experiences can be considered victimization (Mishna et al., 2006).

Victimization among SGD youth may thus be congruently recognized, but also incongruently by youth or adults only. As suggested by the minority stress framework (Brooks, 1981; Meyer, 1995, 2003) and the generalized unsafety theory of stress (Diamond & Alley, 2022), stigma experienced by SGD youth contributes to higher rates of and more persistent exposure to victimization, as well as negative interpersonal relationships and heightened hypervigilance. These factors contribute to distinct patterns of victimization among SGD youth in three different ways: only recognized by youth, only recognized by parents/teachers, and congruently recognized by both youth and adults.

Regarding recognition by youth but not parents or teachers, it is possible that negative parent–child or teacher–student relationships can limit some SGD youth's disclosure, which can limit adults' capability to detect youth's victimization (Goodman et al., 2010). Moreover, stigmatizing experiences might make SGD youth hypervigilant and more sensitive to noticing subtle victimization. Previous research has revealed that victimized youth showed an increased sensitivity to social threats (Schacter et al., 2024). In this case, SGD youth themselves may report peer victimization that may be less likely to be recognized by parents or teachers.

Regarding recognition by parents and teachers but not youth, youth with previous victimization experiences may normalize victimization as their coping strategy (Tremblay‐Perreault et al., 2022). When victimization is prevalent, youth may consider it the norm (Mishna et al., 2006). SGD youth are likely to experience persistent and pervasive victimization that emerges already in childhood (Huang et al., 2024), which may make some SGD youth normalize victimization and lead to situations where victimization is recognized by parents or teachers but not by youth themselves.

Peer victimization among SGD youth may also be congruently recognized. SGD youth tend to experience more severe and persistent victimization (Huang et al., 2024; Kaufman et al., 2020; Martin‐Storey & Fish, 2019). Youth tend to disclose severe cases to adults (Mishna et al., 2006), and the higher severity potentially makes these incidents more visible to others. In this case, youth and adults (parents and teachers) would recognize victimization congruently. In sum, via different theoretical mechanisms, there might be mixed patterns in how SGD youth's victimization is recognized by youth, parents, and teachers.

Previous research on SGD youth's victimization did not directly combine cross‐informant reports, yet multi‐informant studies revealed mixed incongruence among youth, parents, and teachers. While SGD youth reported higher rates of victimization compared to HC youth, parents tended to underestimate (Kaufman et al., 2020) or even failed to detect (Martin‐Storey & Fish, 2019) such victimization disparities, and teachers identified these disparities during late childhood (ages 9–10) but not in adolescence (Martin‐Storey & Fish, 2019). Therefore, it is worth combining parent–youth and teacher–youth reports to discover potential SGD‐based disparities in cross‐informant patterns.

These disparities might change with youth development. Indeed, the development of SGD‐based disparities in report patterns could coincide with the development of sexuality and gender identity during adolescence (Baams & Kaufman, 2023). While youth can be aware of their non‐heterosexual attraction during childhood, most sexually diverse youth can better understand and label their sexual identity in adolescence (Martos et al., 2015; Mustanski et al., 2014). For gender diverse youth, adolescence is a challenging period as they encounter greater discomfort in facing their physiological development (Bockting, 2014; Toomey, 2021). These psychological and physiological changes seem to match the trajectory of victimization, as victimization among SGD youth was found to become salient starting from adolescence (Huang et al., 2024).

### Victimization patterns and youth mental health

Beyond victimization itself, how it is recognized by youth and adults is also linked to youth's mental health. Different recognition patterns may be linked to youth's mental health through different mechanisms. According to the Discrepancies in Victimization Implicate Developmental Effects (DiVIDE) framework (Goodman et al., 2010), when victimization is not sufficiently recognized by adults, victimized youth may develop maladaptive coping without adults' support, which might result in worse adjustment. In contrast, victimization perceived by adults but not by youth does not necessarily mean a false alarm but could reflect youth's hindered capability to identify victimization (Tremblay‐Perreault et al., 2022).

Empirical studies on the role of cross‐informant patterns, based on general populations, revealed that all victimization recognition patterns, including incongruent ones, were linked to worse adjustment (Goodman, 2013; Tremblay‐Perreault et al., 2022). However, findings were mixed regarding which pattern was associated with the worst outcomes, and most findings did not support the DiVIDE framework. For instance, Tremblay‐Perreault et al. (2022) combined frequency of peer victimization reported by general school‐aged children (four items) and their parents (five items), with no timeframe specified for either informant. One non‐victim group and three victimized subgroups were found: congruent victims, parent–identified victims, and youth‐identified victims, of which the congruent victims had the poorest behavioral and social adjustment after six months. Goodman (2013) combined the frequency of interpersonal violence victimization reported by general youth aged 9–12 years and their parents. Both youth and parents reported on the youth's victimization experiences in the past year using the same items. Again, three victimized subgroups were identified: a group with relative agreement (both parent and youth reported low to moderate levels of victimization), a group with parents reporting more victimization than youth, and a group with youth reporting more victimization than parents. Youth in the group where parents reported more victimization than youth displayed the highest levels of internalizing symptoms after a 2.5‐year follow‐up. It should be noted that youth in the relative agreement group in Goodman's study displayed only mild to moderate levels of victimization, while the congruent victims identified by Tremblay‐Perreault et al. showed the highest level of victimization among the sample. Thus, the severity of victimization may act as a confounding factor, which should be controlled for when examining the association between report patterns and mental health outcomes.

### Explaining SGD youth's mental health disparities through cross‐informant victimization reports

As studies among general youth have shown that different cross‐informant patterns are associated differently with youth's adjustment, we argue that, beyond peer victimization experiences, how victimization is recognized might explain SGD youth's mental health disparities. With marginalized identities and potential psychosocial vulnerabilities, SGD youth may be more likely than their HC peers to experience victimization, regardless of whether it was recognized by youth alone, adults alone, or both. These different recognition patterns may further contribute to worse mental health among SGD youth beyond the frequency of victimization. Although previous studies linked SGD youth's adjustment disparities to either youth or adults' reports of victimization (Huang et al., 2024; Kaufman et al., 2020), it remains unclear whether the victimization was recognized by other informants, and whether such recognition, or lack thereof, could help explain their mental health disparities. Comparing all patterns of victimization to the pattern of non‐victimization, we aimed to answer which victimization patterns can explain SGD‐based victimization disparities and their correlated mental health discrepancies. Specifically, this allowed us to assess whether incongruently recognized victimization, compared to non‐victimization, can explain associations between SGD status and mental health, which could also help understand the value of reports from a single informant. Furthermore, comparing congruently and incongruently recognized victimization allows us to examine if victimization among SGD youth is more or less likely to be congruently reported, and how the (in)congruent recognitions, beyond victimization frequency, would contribute to associations between SGD status and mental health.

Sexual orientation is a gradient concept, and some people may identify as mainly heterosexual or report being mostly other‐sex‐attracted. For these individuals, the deviance from the heteronormative social norm is less visible. Mainly heterosexual youth displayed distinct sexuality compared to exclusively heterosexual and bisexual youth (Savin‐Williams & Vrangalova, 2013; Thompson & Morgan, 2008). Relatedly, they were less exposed to minority stress than other sexually diverse groups, with their adjustment falling between exclusively heterosexual and bisexual individuals (Huang et al., 2024; Vrangalova & Savin‐Williams, 2014). Therefore, lumping the mainly heterosexual group with either exclusively heterosexual youth or sexually diverse youth could obscure their distinct experiences and could also lead to an underestimation of sexually diverse youth's disparities. We therefore considered the mainly heterosexual group as a distinct group, separate from the exclusively heterosexual group and the other sexually diverse groups.

Victimization disparities between different identity groups can reinforce the link between marginalized identities and stigmatized social status, and identity‐based bullying is a well‐established stressor for youth's health and well‐being (Bayram Özdemir et al., 2024; Lambe et al., 2025). Stigma and bias translate into youth's negative outcomes through a pathway that involves multiple steps, each of which stands as an opportunity to provide support and promote healthier development. For SGD youth, stigma and bias may shape not only how frequently and severely victimization occurs, but also how these experiences are perceived and recognized by youth and the adults around them. This recognition process is particularly relevant, as recognition serves as the precondition for recovery and intervention. From a global perspective, such recognition may depend on broader social norms, policies, and levels of acceptance of sexual and gender diversity. By examining these processes in the United Kingdom, a context that has undergone substantial advances in SGD rights while victimization and mental health disparities persist (Stonewall, n.d.; Huang et al., 2024; ILGA‐Europe, 2026), the present study explored recognition as an additional social process through which bias may become linked to youth outcomes. Although situated within a single national context, elucidating this process can inform broader theories and provide a basis for future cross‐context comparison.

### Current study

The current study utilized a British sample to explore victimization disparities for SGD youth. Since the turn of the 21st century, the United Kingdom has witnessed significant political changes for SGD individuals (Stonewall, n.d.): Section 28, which prohibited the promotion of homosexuality, was repealed in 2000–2003, civil partnerships for same‐sex couples were introduced in 2004, and the legal recognition of same‐sex marriage was legalized in 2013. Additionally, the Gender Recognition Act of 2004 enabled legal recognition for transgender individuals, and the Equality Act of 2010 established legal protections against discrimination based on sexual orientation and gender reassignment. With these progressive shifts, the United Kingdom ranked first on the European Rainbow Map Index between 2013 and 2015 and maintained a top‐three position until 2019 (ILGA‐Europe, 2026). However, substantial victimization and mental health disparities persist between British SGD youth and their HC peers. We examined victimization disparities for British SGD youth based on the combination of parent–youth‐ and teacher–youth‐reported victimization, and also tested how such disparities would be related to mental health discrepancies between SGD youth and HC youth with two research questions (RQs). Given that parents and teachers have different perspectives on recognizing youth's peer victimization, their recognition and congruence with youth reports were tested separately.

First, we examined whether SGD youth were more likely than HC youth to be recognized as victimized, and whether different victimization‐involved patterns would be linked to worse mental health (RQ1). Specifically, we compared non‐victimized youth to those who were victimized according to: (1) only‐youth‐perceived, (2) only‐adult‐perceived, or (3) congruently recognized. We expected that SGD youth would be overrepresented in all victimized categories, reflecting their elevated risk and the unique informant dynamics theorized in previous research. Given the limited empirical support for the DiVIDE framework, we only used it as a motivational background for the current study. Instead, we broadly expected that victimization, no matter who recognized it, would be related to SGD youth's worse mental health compared to HC youth. Second, among youth who were recognized as victimized, we examined whether victimization among SGD youth (compared to HC youth) was more likely to be congruently or incongruently recognized, and how this was related to worse mental health (RQ2). That is, we compared incongruent reports (only‐youth or only‐adult‐perceived) and congruent reports (perceived by both youth and adults). Given the limited prior work on this topic and mixed findings in general populations, we did not formulate specific hypotheses for this part of the analyses.

Additionally, we aimed to examine cross‐informant victimization patterns across different developmental stages, at ages 7, 11, and 14 years. We expected disparities in victimization patterns to emerge around the transition into adolescence (ages 11 onwards). Further, we aimed not only to test the role of victimization patterns on youth's concurrent mental health, but also to test the role of victimization patterns on follow‐up mental health after a 3–4 year interval. We expected that victimization patterns would be linked to SGD youth's worse mental health both concurrently and longitudinally. The current design and hypotheses were preregistered (https://osf.io/ekabu/?view_only=8ebdb18a4b4e41e5b80f6ae94e349ac2).

## METHODS

### Participants

This study utilized the sample of the Millennium Cohort Study (MCS) project, a national representative, multi‐informant longitudinal study in the United Kingdom. Families with children born in 2000–2002 were randomly selected with a stratified design to cover the disadvantaged and ethnically diverse areas. In total, 19,244 families participated in the MCS project. The current study used data from Wave 4 (W4, 2008, youth aged 7 years), Wave 5 (W5, 2012, youth aged 11 years), Wave 6 (W6, 2015, youth aged 14 years), and Wave 7 (W7, 2018, youth aged 17 years) (Connelly & Platt, 2014). To be specific, parents' (W4–W6), teachers' (W4–W5), and youth's (W4–W6) reports on victimization, together with youth‐reported mental health (W4–W7), were analyzed. Youth, parents, and teachers were reached via home visits, postal, and web surveys. Participants' consent was required at each wave. The secondary analysis of the MCS data was approved by the Ethics Committee of the Faculty of Social and Behavioral Sciences of Utrecht University. In the MCS project, sexual orientation and gender identity were first measured at W7 (age 17), where 10,106 youth had at least one valid response on SGD status. To address disparities between SGD and HC youth, our study linked the SGD status reported at W7 retrospectively to earlier waves, which is a common research strategy addressing prospective development among SGD youth (Kaufman et al., 2020; Martin‐Storey & Fish, 2019).

Based on the study aims, youth who had at least one valid response on SGD status and who had at least one pair of victimization reported by parent–youth or teacher–youth dyads were selected as the analytical sample (see Figure S1 for a flow diagram). For each parent–youth and teacher–youth model, pairwise deletion was used to examine cross‐informant patterns in victimization reports. For parent–youth‐reported victimization, 8260 dyads at W4, 8846 dyads at W5, and 8755 dyads at W6 were analyzed. For teacher–youth‐reported victimization, 5708 dyads at W4 and 5354 dyads at W5 were analyzed. Relatively smaller sample sizes for teacher–youth dyads were owing to less valid teacher‐reported data in the MCS project. In total, *n* = 9936 cases (51.3% assigned female at birth) were selected, among whom 78.6% were White, 3.4% were mixed, 2.9% were Indian, 7.9% were Pakistani and Bangladeshi, 4.1% were Black or Black British, and 3.1% were other ethnic groups. Besides, 28.9% of youth were born in 2000, 70.6% in 2001, and 0.5% in 2002.

### Measurement

#### Sexual orientation

Youth self‐reported sexual orientation was assessed at W7 by two items. The sexual identity item “Which of the following options best describes how you currently think of yourself?” was measured with 8 options: 1 = Completely heterosexual/straight, 2 = Mainly heterosexual/straight, 3 = Bisexual, 4 = Mainly gay or lesbian, 5 = Completely gay or lesbian, 6 = Other, 7 = Don't know, and 8 = Prefer not to say (coded as missing). The sexual attraction item “I have felt sexually attracted…” was measured with 6 options: 1 = Only to the opposite sex and never to the same sex, 2 = More often to the opposite sex and at least once to the same sex, 3 = About equally often to the opposite sex and to the same sex, 4 = More often to the same sex and at least once to the opposite sex, 5 = Only ever to the same sex and never to the opposite sex, and 6 = I have never felt sexually attracted to anyone at all.

Sexual identity and attraction are two distinct essential dimensions of sexual orientation. Studies showed that about 99% of people who are attracted only to the opposite sex would self‐identify as heterosexual (Copen et al., 2016). Thus, for model simplicity, we integrated these two dimensions while maximizing the identification of sexually diverse youth. First, youth who reported [3 = about equally often to the opposite sex and to the same sex, 4 = More often to the same sex and at least once to the opposite sex, 5 = Only ever to the same sex and never to the opposite sex, and 6 = I have never felt sexual attracted to anyone at all] on the sexual attraction item, or identified as [3 = Bisexual, 4 = Mainly gay or lesbian, 5 = Completely gay or lesbian, 6 = Other, 7 = Don't know] on the sexual identity items, were coded as *sexually diverse* (*n* = 1296, 13.0%). Among the rest of the participants, youth who reported [2 = More often to the opposite sex and at least once to the same sex] on the sexual attraction item, or identified as [2 = Mainly heterosexual/straight] were coded as *mainly heterosexual* (*n* = 1377, 13.9%). All the remaining participants with valid answers were coded as *exclusively heterosexual* (*n* = 7240, 72.9%). Youth without valid answers on the sexual orientation items were coded as missing (*n* = 23).

#### Sex assigned at birth

Parents reported youth's sex assigned at birth, which was coded as 0 = male and 1 = female.

#### Gender identity

Youth self‐reported gender identity was measured at W7 by one item “Which of the following options best describes how you currently think of yourself?” with 8 options: 1 = Male, 2 = Female, 3 = Other, 4 = Androgynous, 5 = Gender fluid, 6 = Non‐binary, 7 = Don't know, and 8 = Prefer not to say (coded as missing). Youth who identified as the opposite gender in comparison with their sex assigned at birth, and who reported [3 = Other, 4 = Androgynous, 5 = Gender fluid, 6 = Non‐binary, 7 = Don't know] on the gender identity item, were coded as *gender diverse* (*n* = 108, 1.1%). Youth whose gender identity was in line with their assigned sex were coded as *cisgender* (*n* = 9771, 98.3%). Youth without a valid answer on the gender identity item were coded as missing (*n* = 57).

#### Peer victimization

Youth‐reported peer victimization was measured by one item at W4 (“How often do other children bully you,” 1 = never to 3 = all of the time), and one item at W5 and W6 (“How often do other children hurt you or pick on you,” 1 = never to 6 = most days). No definition of bullying was provided prior to the items. With the present tense used in the item, no timeframe was specified.

Parents/Teachers‐reported peer victimization was measured using one item extracted from the Strengths and Difficulties Questionnaire (SDQ) (Goodman & Goodman, 2011). Parents and teachers were asked to recall the youth's behavior over the last six months: “[Kid's name] is picked on or bullied by other children” (1 = not true to 3 = very true). No definition of bullying was provided prior to the items. Parent‐reported data from W4 to W6 and teacher‐reported data at W4 and W5 were used. To assess cross‐informant victimization patterns, all the victimization items were coded as 0 = never/not true and 1 = all other scores, while the original scores of youth‐reported frequency were used for severity control.

#### Emotional problems

Youth self‐reported emotional problems were measured from W4 to W7.

W4 emotional problems were measured with seven moods (e.g., “How often do you feel sad,” 1 = never to 3 = all of the time). Confirmatory factor analysis (CFA) was conducted, and three items were dropped due to low factor loading. Although the Cronbach's alpha for W4 was .45, the items revealed a satisfactory factor structure (Appendix S1, Table S1).

W5 emotional problems were measured with six moods (e.g., “How often do you feel sad,” 1 = never to 5 = almost always). CFA was conducted, and one item was dropped due to low factor loading (Table S1). The Cronbach's alpha for W5 was .73.

W6 emotional problems were measured using the 13‐item Short Mood and Feelings Questionnaire (SMFQ; e.g., “In the past two weeks I felt miserable or unhappy,” 1 = Not true to 3 = True) (Angold et al., 1995). The Cronbach's alpha for W6 was .93.

W7 emotional problems were measured using the 6‐item Kessler 6 scale (K6; e.g., “During the last 30 days, about how often did you feel hopeless,” 1 = none of the time to 5 = all of the time) (Kessler et al., 2002). The Cronbach's alpha for W7 was .86.

The mean scores for each emotional problems measure were computed for each wave separately for further analysis.

#### Self‐esteem

Self‐esteem was measured from W5 to W7 by five items extracted from the Rosenberg self‐esteem scale (e.g., “I feel good about myself”) (Rosenberg, 1989). Youth reported on a 4‐point scale from 1 = Strongly disagree to 4 = Strongly agree. The Cronbach's alpha for W5 to W7 ranged from .74 to .91. The mean scores were used for further analysis.

### Data analysis

Statistical models were tested using Mplus (version 8.9). To handle models with missing data, full information maximum likelihood (FIML) analyses in Mplus were employed for all model estimations, with maximum likelihood with robust standard errors (MLR) specified as the estimator to model non‐normal data. Indirect effects were estimated using the MODEL CONSTRAINT command in Mplus, calculated by the products of the path from SGD status to victimization classes and the path from victimization classes to mental health outcomes. Montecarlo integration was specified to handle models with categorical data. Limited by the distribution of data, the pre‐registered latent class approach was not feasible (see Appendix S2 for details). Thus, youth were manually classified. Combining youth and parent reports (W4–W6, each wave separately), youth were classified as: *non‐victims* (neither youth nor parents reported victimization), *only‐youth‐perceived victims* (only youth but not parents reported victimization), *only‐parent‐perceived victims* (only parents but not youth reported victimization), and *congruent victims* (both youth and parents reported victimization). Similarly, combining youth and teacher reports (W4–W5, each wave separately), youth were classified as: *non‐victims, only‐youth‐perceived victims, only‐teacher‐perceived victims*, and *congruent victims*.

To explore whether SGD status was related to the victimization classes, and whether victimization classes were associated with mental health disparities for SGD youth, path analysis with indirect effects was applied, with victimization classes assigned as the mediator between SGD status and youth's mental health (emotional problems and self‐esteem). To examine concurrent and longitudinal associations, both concurrent (W_*n*_) and follow‐up (W_*n*+1_) mental health indicators were included in the same model as outcomes, with the follow‐up mental health controlled for concurrent ones.

The indirect analyses proceeded in two steps. First, *victim* versus *non‐victim models* (Figure 1a) were specified to discover whether any victimization pattern, recognized congruently or incongruently, would be related to SGD youth's worse mental health. In these models, the memberships of the three victimized classes were assigned as parallel mediators in the same model. In doing so, the non‐victim class was set as the reference, which allowed us to address the mental health discrepancies between victimized and non‐victimized youth.

**FIGURE 1:**
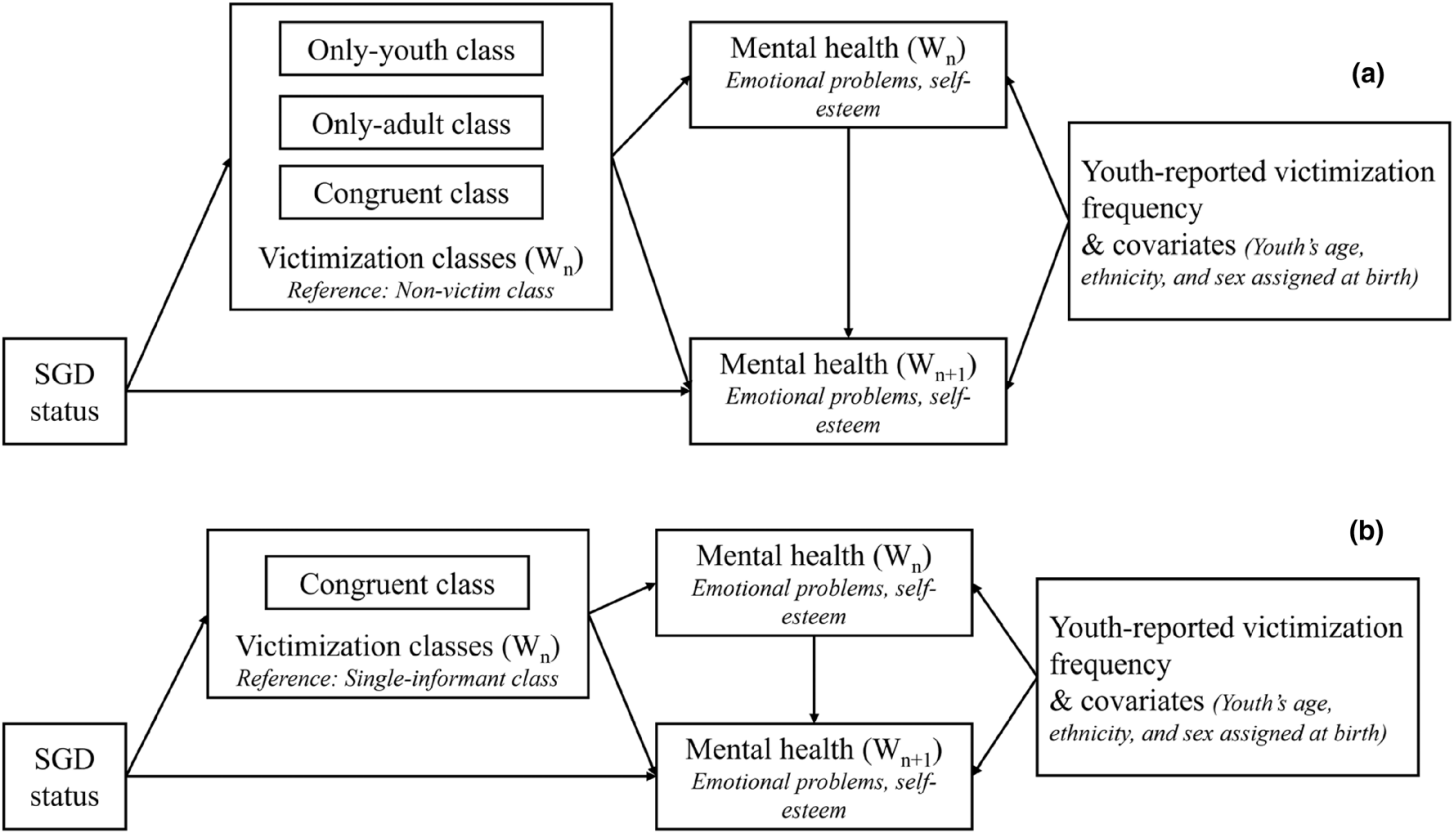
Victim vs non‐victim model (a), and congruent vs single‐reporter model (b). SGD, sexual and gender diverse; W, wave.

Second, to understand the role of congruent recognition among victimized youth, we compared congruently identified victims with those identified solely by one source. Therefore, in the *congruent* versus *single‐reporter model* (Figure 1b), the mediator was coded as congruent victims = 1 and single‐informant‐perceived victims = 0 (i.e., only‐youth‐perceived, only‐parent‐perceived, or only‐teacher‐perceived victims, each in a separate model).

Given that previous findings emphasized the importance of severity, the indirect models were adjusted for youth's self‐reported concurrent frequency of victimization. Adult reports were not controlled because they reflected awareness rather than the actual severity of victimization. The role of sexual orientation and gender identity was tested in separate models. For sexual‐orientation‐based disparities, three subgroups were created and tested in the same model; the role of being sexually diverse and mainly heterosexual was both tested, setting the exclusively heterosexual youth as the reference group. For gender‐identity‐based disparities, two subgroups were created, and the role of being gender diverse was tested, setting the cisgender youth as the reference group. Youth's age, ethnicity (coded as 0 = white and 1 = other ethnic groups), and sex assigned at birth were controlled for in the models. Missingness in covariates was handled by estimating the variances.

A reflexivity statement describing the authors' positionality and how our backgrounds informed this study is provided in the Appendix S3.

## RESULTS

### Preliminary analyses

Missingness analyses comparing included and excluded participants are presented in Appendix S4: Tables S2–S4. Descriptive statistics and correlations are presented in Appendix S5 (Table S5). ANOVA results (Appendix S6, Table S6) indicated that youth in the congruent victims class reported significantly higher victimization frequency compared to those in the only‐youth‐perceived class. This finding underscores the need to control for youth‐reported victimization frequency when examining links to mental health.

Regarding the proportion of victimization classes and SGD youth's distribution (Table 1), most victimized youth were classified as only‐youth‐perceived victims, suggesting a general underestimation of youth's peer victimization by both parents and teachers. Due to sample size constraints, indirect models for gender diverse youth were only tested for: (1) victim versus non‐victim based on parent–youth reports, and (2) congruent versus only‐youth‐perceived based on parent–youth reports.

**TABLE 1:** Sexual and gender diverse youth distribution in cross‐informant victimization classes.

SGD status			Parent–youth reports											
			Wave 4				Wave 5				Wave 6			
			Non	Youth	Parent	Congruent	Non	Youth	Parent	Congruent	Non	Youth	Parent	Congruent
SO	Exclusively heterosexual	*n*	2794	2083	350	775	2537	2472	326	1109	2997	2082	453	820
		%	46.6%	34.7%	5.8%	12.9%	39.4%	38.4%	5.1%	17.2%	47.2%	32.8%	7.1%	12.9%
	Mainly heterosexual	*n*	547	406	66	151	426	537	53	235	424	562	82	182
		%	46.8%	34.7%	5.6%	12.9%	34.1%	42.9%	4.2%	18.8%	33.9%	45.0%	6.6%	14.6%
	Sexually diverse	*n*	467	347	71	183	310	430	78	318	330	406	98	306
		%	43.7%	32.5%	6.6%	17.1%	27.3%	37.9%	6.9%	28.0%	28.9%	35.6%	8.6%	26.8%
Total		*n*	3808	2836	487	1109	3273	3439	457	1662	3751	3050	633	1308
		%	46.2%	34.4%	5.9%	13.5%	37.1%	38.9%	5.2%	18.8%	42.9%	34.9%	7.2%	15.0%
GI	Cisgender	*n*	3759	2793	484	1086	3242	3391	448	1621	3728	2985	627	1259
		%	46.3%	34.4%	6.0%	13.4%	37.3%	39.0%	5.1%	18.6%	43.4%	34.7%	7.3%	14.6%
	Gender diverse	*n*	45	30	1	17	27	34	3	31	19	44	5	37
		%	48.4%	32.3%	1.1%	18.3%	28.4%	35.8%	3.2%	32.6%	18.1%	41.9%	4.8%	35.2%
Total		*n*	3804	2823	485	1103	3269	3425	451	1652	3747	3029	632	1296
		%	46.3%	34.4%	5.9%	13.4%	37.2%	38.9%	5.1%	18.8%	43.0%	34.8%	7.3%	14.9%
**SGD status**			**Teacher–youth reports**											
			**Wave 4**				**Wave 5**							
			**Non**	**Youth**	**Teacher**	**Congruent**	**Non**	**Youth**	**Teacher**	**Congruent**				
SO	Exclusively heterosexual	*n*	2002	1806	107	166	1566	1912	103	299				
		%	49.1%	44.3%	2.6%	4.1%	40.4%	49.3%	2.7%	7.7%				
	Mainly heterosexual	*n*	412	379	25	39	270	444	18	67				
		%	48.2%	44.3%	2.9%	4.6%	33.8%	55.6%	2.3%	8.4%				
	Sexually diverse	*n*	358	333	27	44	202	348	17	99				
		%	47.0%	43.7%	3.5%	5.8%	30.3%	52.3%	2.6%	14.9%				
Total		*n*	2772	2518	159	249	2038	2704	138	465				
		%	48.6%	44.2%	2.8%	4.4%	38.1%	50.6%	2.6%	8.7%				
GI	Cisgender	*n*	2726	2475	158	246	2023	2662	134	455				
		%	48.6%	44.2%	2.8%	4.4%	38.4%	50.5%	2.5%	8.6%				
	Gender diverse	*n*	39	32	2	2	15	32	3	11				
		%	52.0%	42.7%	2.7%	2.7%	24.6%	52.5%	4.9%	18.0%				
Total		*n*	2765	2507	160	248	2038	2694	137	466				
		%	48.7%	44.1%	2.8%	4.4%	38.2%	50.5%	2.6%	8.7%				

Abbreviations: Cong, congruent victim class; GI, gender identity; Non, non‐victim class; Parent, only‐parent‐perceived class; SGD, sexual and gender diverse; SO, sexual orientation; Teacher, only‐teacher‐perceived class; Youth, only‐youth‐perceived class.

### RQ1: Victimization classes compared with the non‐victimization class

The first research question examined whether SGD youth were more likely than HC youth to belong to victimization‐involved classes (only‐youth, only‐adult, or congruent) rather than the non‐victim class, and whether these victimization patterns were associated with concurrent and follow‐up mental health. Full results for main and controlled effects are in Appendix S7 (Tables S7 and S8).

#### Sexually diverse youth

For *sexually diverse* youth, results based on parent–youth reports (Table 2) showed they were more likely than *exclusively heterosexual* peers to be in the congruent class at all tested ages (7–14) and in the only‐parent class at age 11. They were not more likely to be in the only‐youth class. These victimization patterns were concurrently associated with worse mental health. Moreover, congruently recognized victimization at ages 11–14 was related to *sexually diverse* youth's worse follow‐up mental health (ages 14–17).

**TABLE 2:** Victim vs. non‐victim model: parent–youth reports with sexual orientation status.

Sexually diverse youth (exclusively heterosexual as reference class)										
Paths	Emotional problems						Self‐esteem			
	Wave 4 (Age 7)		Wave 5 (Age 11)		Wave 6 (Age 14)		Wave 5 (Age 11)		Wave 6 (Age 14)	
	*b*/*β*	95% CI	*b*/*β*	95% CI	*b*/*β*	95% CI	*b*/*β*	95% CI	*b*/*β*	95% CI
SDiv. → Cong	0.33***	[0.16, 0.51]	0.63***	[0.48, 0.77]	0.91***	[0.76, 1.06]	0.63***	[0.48, 0.77]	0.91***	[0.76, 1.06]
Cong → MH (W_*n*_)	0.06**	[0.02, 0.11]	0.18***	[0.13, 0.23]	0.12***	[0.09, 0.16]	−0.1***	[−0.14, −0.07]	−0.17***	[−0.22, −0.13]
Cong → MH (W_*n*+1_)	0.05	[−0.04, 0.14]	0.07***	[0.04, 0.11]	0.12***	[0.06, 0.18]	−0.1***	[−0.14, −0.05]	−0.06*	[−0.11, −0.01]
SDiv. → Cong → MH (W_*n*_)	0.021*	[0.003, 0.039]	0.115***	[0.073, 0.156]	0.111***	[0.073, 0.149]	−0.064***	[−0.09, −0.037]	−0.155***	[−0.204, −0.107]
SDiv. → Cong → MH (W_*n*+1_)	0.016	[−0.016, 0.048]	0.046***	[0.021, 0.071]	0.108***	[0.049, 0.168]	−0.059***	[−0.091, −0.028]	−0.053*	[−0.1, −0.007]
SDiv. → Youth	−0.1	[−0.24, 0.04]	−0.02	[−0.15, 0.11]	0.13	[−0.01, 0.26]	−0.02	[−0.15, 0.11]	0.13	[−0.01, 0.26]
Youth → MH (W_*n*_)	0.04*	[0.01, 0.08]	0.09***	[0.06, 0.13]	0.07***	[0.04, 0.09]	−0.04**	[−0.07, −0.01]	−0.09***	[−0.12, −0.06]
Youth → MH (W_*n*+1_)	−0.05	[−0.13, 0.02]	0.03*	[0, 0.06]	0.09***	[0.05, 0.13]	−0.04*	[−0.07, 0]	−0.04*	[−0.07, 0]
SDiv. → Youth → MH (W_*n*_)	−0.004	[−0.011, 0.003]	−0.002	[−0.014, 0.01]	0.009	[−0.001, 0.018]	0.001	[−0.004, 0.006]	−0.011	[−0.024, 0.001]
SDiv. → Youth → MH (W_*n*+1_)	0.005	[−0.005, 0.016]	−0.001	[−0.005, 0.003]	0.011	[−0.002, 0.025]	0.001	[−0.004, 0.006]	−0.005	[−0.011, 0.002]
SDiv. → Parent	0.14	[−0.12, 0.4]	0.33*	[0.07, 0.58]	0.21	[−0.02, 0.43]	0.33*	[0.07, 0.58]	0.21	[−0.02, 0.43]
Parent → MH (W_*n*_)	0.01	[−0.02, 0.04]	0.12***	[0.06, 0.18]	0.08***	[0.05, 0.12]	−0.08***	[−0.12, −0.04]	−0.05*	[−0.1, −0.01]
Parent → MH (W_*n*+1_)	0.09**	[0.03, 0.15]	0.04	[−0.01, 0.08]	0.09**	[0.03, 0.16]	−0.04	[−0.09, 0.02]	−0.06*	[−0.11, −0.01]
SDiv. → Parent → MH (W_*n*_)	0.001	[−0.004, 0.007]	0.039*	[0.002, 0.075]	0.017	[−0.003, 0.036]	−0.026*	[−0.051, −0.001]	−0.011	[−0.026, 0.005]
SDiv. → Parent → MH (W_*n*+1_)	0.012	[−0.012, 0.037]	0.012	[−0.005, 0.029]	0.019	[−0.006, 0.044]	−0.013	[−0.033, 0.008]	−0.012	[−0.029, 0.005]
Mainly heterosexual youth (Exclusively heterosexual as reference class)										
Main → Cong	0	[−0.19, 0.19]	0.11	[−0.05, 0.26]	0.14	[−0.03, 0.31]	0.11	[−0.05, 0.26]	0.14	[−0.03, 0.31]
Main → Cong → MH (W_*n*_)	0	[−0.012, 0.012]	0.02	[−0.009, 0.049]	0.017	[−0.005, 0.039]	−0.011	[−0.027, 0.005]	−0.024	[−0.054, 0.006]
Main → Cong → MH (W_*n*+1_)	0	[−0.009, 0.009]	0.008	[−0.004, 0.02]	0.017	[−0.006, 0.039]	−0.01	[−0.026, 0.006]	−0.008	[−0.021, 0.004]
Main → Youth	0	[−0.13, 0.13]	0.19**	[0.07, 0.31]	0.52***	[0.39, 0.64]	0.19**	[0.07, 0.31]	0.52***	[0.39, 0.64]
Main → Youth → MH (W_*n*_)	0	[−0.005, 0.005]	0.018**	[0.004, 0.031]	0.036***	[0.02, 0.051]	−0.007*	[−0.014, −0.001]	−0.046***	[−0.066, −0.025]
Main → Youth → MH (W_*n*+1_)	0	[−0.007, 0.007]	0.006	[−0.001, 0.012]	0.047***	[0.022, 0.072]	−0.007	[−0.015, 0.001]	−0.019	[−0.037, 0]
Main → Parent	−0.03	[−0.3, 0.24]	−0.19	[−0.48, 0.11]	−0.09	[−0.33, 0.16]	−0.19	[−0.48, 0.11]	−0.09	[−0.33, 0.16]
Main → Parent → MH (W_*n*_)	0	[−0.003, 0.003]	−0.022	[−0.059, 0.015]	−0.007	[−0.027, 0.013]	0.015	[−0.01, 0.039]	0.005	[−0.009, 0.018]
Main → Parent → MH (W_*n*+1_)	−0.003	[−0.027, 0.021]	−0.007	[−0.02, 0.007]	−0.008	[−0.031, 0.015]	0.007	[−0.008, 0.023]	0.005	[−0.01, 0.02]

*Note*: In this table, the direct effect estimates are presented as standardized coefficients (*β*), while the indirect effect estimates are reported as unstandardized coefficients (*b*).

Abbreviations: CI, confidence interval; Cong, congruent victim; Main, mainly heterosexual; MH, mental health outcomes; Parent, only‐parent‐perceived class; SDiv, sexually diverse; Youth, only‐youth‐perceived class.

* *p* < .05.

** *p* < .01.

*** *p* < .001.

Teacher–youth reports (Table 3) revealed a similar pattern: *Sexually diverse* youth were more likely than *exclusively heterosexual* peers to be congruently recognized as victims at both tested ages (7 and 11). This congruence was associated with worse mental health concurrently at age 11, but not at age 7. No longitudinal associations were found. No significant differences emerged for the only‐youth or only‐teacher class.

**TABLE 3:** Victim vs. non‐victim model: teacher–youth reports with sexual orientation status.

Sexually diverse youth (exclusively heterosexual as reference class)						
Paths	Emotional problems				Self‐esteem	
	Wave 4 (Age 7)		Wave 5 (Age 11)		Wave 5 (Age 11)	
	*b*/*β*	95% CI	*b*/*β*	95% CI	*b*/*β*	95% CI
SDiv. → Cong	0.37*	[0.03, 0.71]	0.74***	[0.5, 0.98]	0.74***	[0.5, 0.98]
Cong → MH (W_*n*_)	0.07*	[0.01, 0.12]	0.2***	[0.12, 0.27]	−0.07*	[−0.13, −0.02]
Cong → MH (W_*n*+1_)	0.13*	[0, 0.26]	0.03	[−0.03, 0.08]	−0.03	[−0.09, 0.04]
SDiv. → Cong → MH (W_*n*_)	0.024	[−0.008, 0.055]	0.144***	[0.068, 0.22]	−0.054*	[−0.098, −0.009]
SDiv. → Cong → MH (W_*n*+1_)	0.049	[−0.018, 0.115]	0.019	[−0.021, 0.059]	−0.019	[−0.069, 0.032]
SDiv. → Youth	−0.02	[−0.18, 0.13]	0.12	[−0.05, 0.28]	0.12	[−0.05, 0.28]
Youth → MH (W_*n*_)	0.04	[−0.01, 0.08]	0.1***	[0.06, 0.15]	−0.05**	[−0.08, −0.01]
Youth → MH (W_*n*+1_)	−0.02	[−0.11, 0.07]	0.03	[0, 0.07]	−0.05*	[−0.09, −0.01]
SDiv. → Youth → MH (W_*n*_)	−0.001	[−0.007, 0.005]	0.012	[−0.006, 0.029]	−0.005	[−0.014, 0.003]
SDiv. → Youth → MH (W_*n*+1_)	0	[−0.003, 0.004]	0.004	[−0.003, 0.011]	−0.006	[−0.015, 0.004]
SDiv. → Teacher	0.32	[−0.11, 0.75]	−0.04	[−0.56, 0.48]	−0.04	[−0.56, 0.48]
Teacher → MH (W_*n*_)	−0.01	[−0.07, 0.04]	0.11*	[0.01, 0.22]	0	[−0.08, 0.07]
Teacher → MH (W_*n*+1_)	0.13*	[0.02, 0.25]	0.02	[−0.05, 0.1]	−0.04	[−0.14, 0.07]
SDiv. → Teacher → MH (W_*n*_)	−0.004	[−0.022, 0.014]	−0.005	[−0.063, 0.054]	0	[−0.003, 0.004]
SDiv. → Teacher → MH (W_*n*+1_)	0.042	[−0.026, 0.11]	−0.001	[−0.013, 0.011]	0.001	[−0.018, 0.021]
Mainly heterosexual youth (Exclusively heterosexual as reference class)						
Main → Cong	0.12	[−0.24, 0.48]	0.1	[−0.18, 0.37]	0.1	[−0.18, 0.37]
Main → Cong → MH (W_*n*_)	0.008	[−0.017, 0.032]	0.019	[−0.036, 0.073]	−0.007	[−0.027, 0.014]
Main → Cong → MH (W_*n*+1_)	0.016	[−0.034, 0.066]	0.002	[−0.006, 0.011]	−0.002	[−0.012, 0.007]
Main → Youth	0	[−0.15, 0.15]	0.25**	[0.1, 0.4]	0.25**	[0.1, 0.4]
Main → Youth → MH (W_*n*_)	0	[−0.005, 0.006]	0.025*	[0.006, 0.045]	−0.011*	[−0.022, 0]
Main → Youth → MH (W_*n*+1_)	0	[−0.003, 0.003]	0.008	[−0.002, 0.018]	−0.012	[−0.025, 0.001]
Main → Teacher	0.12	[−0.32, 0.56]	−0.17	[−0.68, 0.34]	−0.17	[−0.68, 0.34]
Main → Teacher → MH (W_*n*_)	−0.001	[−0.01, 0.007]	−0.019	[−0.081, 0.043]	0	[−0.012, 0.013]
Main → Teacher → MH (W_*n*+1_)	0.016	[−0.044, 0.075]	−0.004	[−0.019, 0.012]	0.006	[−0.018, 0.03]

*Note*: In this table, the direct effect estimates are presented as standardized coefficients (*β*), while the indirect effect estimates are reported as unstandardized coefficients (*b*).

Abbreviations: CI, confidence interval; Cong, congruent victim; Main, mainly heterosexual; MH, mental health outcomes; SDiv, sexually diverse; Teacher, only‐teacher‐perceived class; Youth, only‐youth‐perceived class.

* *p* < .05.

** *p* < .01.

*** *p* < .001.

In sum, in both parent–youth and teacher–youth reports, *sexually diverse* youth were consistently likely to be in the congruent class. Congruent recognition by parents and youth was related to worse mental health concurrently and longitudinally, while congruent recognition by teachers and youth was only associated with concurrent mental health. Only one other indirect effect emerged: being in the only‐parent class was associated with worse concurrent mental health at age 11.

#### Mainly heterosexual youth

For *mainly heterosexual* youth, victimization disparities emerged from early adolescence (age 11) onwards. Parent–youth and teacher–youth reports (Tables 2 and 3) both indicated they were more likely to be in the only‐youth class compared to *exclusively heterosexual* youth. This recognition pattern was associated with worse mental health, mostly concurrently. Only one longitudinal effect was found: Being in the only‐youth class at age 14 (in parent–youth reports) was related to greater emotional problems at age 17. No differences emerged in the likelihood of being in the congruent or only‐adult class. In short, *mainly heterosexual* youth were particularly likely to be in the only‐youth class, which was linked to worse mental health mostly concurrently.

#### Gender diverse youth

Results based on parent–youth reports are presented in Table 4. *Gender diverse* youth were more likely to be congruently recognized as victims by themselves and their parents during adolescence compared to cisgender youth (ages 11 and 14, but not 7). This congruence was linked to worse mental health both concurrently and longitudinally. No differences were found for the only‐youth class nor the only‐parent class, though the small number of *gender diverse* youth in the only‐parent class limits statistical power and should be interpreted with caution. In short, *gender diverse* youth were more likely to be in the congruent class, which was linked to worse mental health concurrently and longitudinally.

**TABLE 4:** Victim vs. non‐victim model: parent–youth reports with gender identity status.

Gender diverse youth (cisgender as the reference class)										
Paths	Emotional problems						Self‐esteem			
	Wave 4 (Age 7)		Wave 5 (Age 11)		Wave 6 (Age 14)		Wave 5 (Age 11)		Wave 6 (Age 14)	
	*b*/*β*	95% CI	*b*/*β*	95% CI	*b*/*β*	95% CI	*b*/*β*	95% CI	*b*/*β*	95% CI
GD → Cong	0.37	[−0.16, 0.9]	0.75**	[0.32, 1.18]	1.15***	[0.75, 1.56]	0.75**	[0.32, 1.18]	1.15***	[0.75, 1.56]
Cong → MH (W_*n*_)	0.06**	[0.02, 0.11]	0.19***	[0.14, 0.24]	0.13***	[0.09, 0.17]	−0.1***	[−0.14, −0.07]	−0.18***	[−0.23, −0.13]
Cong → MH (W_*n*+1_)	0.06	[−0.04, 0.15]	0.08***	[0.05, 0.12]	0.13***	[0.07, 0.2]	−0.1***	[−0.15, −0.06]	−0.07*	[−0.12, −0.02]
GD → Cong → MH (W_*n*_)	0.024	[−0.013, 0.061]	0.142**	[0.052, 0.233]	0.149***	[0.081, 0.216]	−0.078**	[−0.13, −0.026]	−0.207***	[−0.298, −0.116]
GD → Cong → MH (W_*n*+1_)	0.021	[−0.025, 0.067]	0.062**	[0.016, 0.107]	0.154**	[0.063, 0.244]	−0.078**	[−0.134, −0.021]	−0.077*	[−0.141, −0.013]
GD → Youth	−0.1	[−0.53, 0.34]	−0.14	[−0.56, 0.29]	0.31	[−0.09, 0.7]	−0.14	[−0.56, 0.29]	0.31	[−0.09, 0.7]
Youth → MH (W_*n*_)	0.04*	[0.01, 0.08]	0.1***	[0.06, 0.13]	0.08***	[0.05, 0.1]	−0.04**	[−0.07, −0.01]	−0.1***	[−0.13, −0.06]
Youth → MH (W_*n*+1_)	−0.05	[−0.13, 0.03]	0.04**	[0.01, 0.06]	0.1***	[0.06, 0.15]	−0.04*	[−0.08, −0.01]	−0.04*	[−0.08, −0.01]
GD → Youth → MH (W_*n*_)	−0.004	[−0.023, 0.015]	−0.013	[−0.055, 0.028]	0.023	[−0.007, 0.054]	0.005	[−0.012, 0.022]	−0.029	[−0.068, 0.009]
GD → Youth → MH (W_*n*+1_)	0.005	[−0.019, 0.029]	−0.005	[−0.021, 0.011]	0.032	[−0.011, 0.074]	0.006	[−0.012, 0.024]	−0.013	[−0.033, 0.007]
GD → Parent	−1.76	[−3.73, 0.21]	−0.51	[−1.66, 0.64]	−0.45	[−1.36, 0.45]	−0.51	[−1.66, 0.64]	−0.45	[−1.36, 0.45]
Parent → MH (W_*n*_)	0.01	[−0.03, 0.04]	0.13***	[0.07, 0.19]	0.09***	[0.06, 0.13]	−0.08**	[−0.12, −0.03]	−0.06**	[−0.11, −0.02]
Parent → MH (W_*n*+1_)	0.1**	[0.03, 0.16]	0.05*	[0, 0.09]	0.12**	[0.05, 0.18]	−0.04	[−0.1, 0.01]	−0.07**	[−0.13, −0.02]
GD → Parent → MH (W_*n*_)	−0.01	[−0.066, 0.047]	−0.065	[−0.215, 0.084]	−0.041	[−0.125, 0.042]	0.038	[−0.05, 0.127]	0.029	[−0.032, 0.09]
GD → Parent → MH (W_*n*+1_)	−0.168	[−0.392, 0.056]	−0.023	[−0.08, 0.034]	−0.052	[−0.159, 0.055]	0.022	[−0.038, 0.083]	0.033	[−0.037, 0.104]

*Note*: In this table, the direct effect estimates are presented as standardized coefficients (*β*), while the indirect effect estimates are reported as unstandardized coefficients (*b*).

Abbreviations: CI, confidence interval; Cong, congruent victim; GD, gender diverse; MH, mental health outcomes; Parent, only‐parent‐perceived class; Youth, only‐youth‐perceived class.

* *p* < .05.

** *p* < .01.

*** *p* < .001.

### RQ2: Congruent recognition compared with single‐informant recognition

The second research question examined whether, among victimized youth, SGD youth were more likely than HC youth to belong to the congruent or single‐informant classes, and whether these class differences were associated with concurrent and follow‐up mental health. Full results for main and controlled effects are in Appendix S7, Tables S9 and S10.

#### Sexually diverse youth

When comparing the youth and parents congruent class with the only‐youth class (Table 5A), *sexually diverse* youth were more likely to be in the congruent class at all tested ages (7–14). The congruent patterns at ages 11–14 were associated with worse concurrent mental health, while the congruent patterns at ages 7–11 were associated with worse follow‐up mental health.

**TABLE 5:** Single informant vs. congruent model with sexual orientation status.

Paths	Emotional problems						Self‐esteem			
	Wave 4 (age 7)		Wave 5 (age 11)		Wave 6 (age 14)		Wave 5 (age 11)		Wave 6 (age 14)	
	*b*/*β*	95% CI	*b*/*β*	95% CI	*b*/*β*	95% CI	*b*/*β*	95% CI	*b*/*β*	95% CI
(A) Only‐youth vs. Congruent (Parent and youth)										
SDiv. → Cong	0.35**	[0.15, 0.55]	0.5***	[0.34, 0.66]	0.65***	[0.48, 0.82]	0.5***	[0.34, 0.66]	0.65***	[0.48, 0.82]
Cong → MH (W_*n*_)	0.02*	[0, 0.04]	0.09***	[0.05, 0.13]	0.05**	[0.02, 0.08]	−0.06***	[−0.09, −0.04]	−0.08***	[−0.12, −0.04]
Cong → MH (W_*n*+1_)	0.1***	[0.05, 0.15]	0.04**	[0.02, 0.07]	0.03	[−0.02, 0.07]	−0.06**	[−0.09, −0.02]	−0.02	[−0.06, 0.02]
SDiv. → Cong → MH (W_*n*_)	0.008	[−0.001, 0.017]	0.045***	[0.02, 0.069]	0.033**	[0.012, 0.054]	−0.032***	[−0.049, −0.015]	−0.052***	[−0.08, −0.025]
SDiv. → Cong → MH (W_*n*+1_)	0.036*	[0.009, 0.062]	0.021**	[0.006, 0.037]	0.016	[−0.015, 0.048]	−0.029**	[−0.049, −0.01]	−0.011	[−0.037, 0.015]
Main → Cong	0	[−0.2, 0.21]	−0.02	[−0.19, 0.14]	−0.2*	[−0.38, −0.01]	−0.02	[−0.19, 0.14]	−0.2*	[−0.38, −0.01]
Main → Cong → MH (W_*n*_)	0	[−0.005, 0.005]	−0.002	[−0.017, 0.013]	−0.01	[−0.021, 0.001]	0.002	[−0.009, 0.012]	0.016	[−0.001, 0.032]
Main → Cong → MH (W_*n*+1_)	0	[−0.021, 0.021]	−0.001	[−0.008, 0.006]	−0.005	[−0.016, 0.006]	0.001	[−0.009, 0.011]	0.003	[−0.005, 0.012]
(B) Only‐parent vs. Congruent (Parent and youth)										
SDiv. → Cong	0.15	[−0.15, 0.45]	0.18	[−0.1, 0.46]	0.54***	[0.29, 0.8]	0.18	[−0.1, 0.46]	0.54***	[0.29, 0.8]
Cong → MH (W_*n*_)	0.07*	[0.01, 0.14]	0.02	[−0.07, 0.1]	0.03	[−0.02, 0.09]	−0.01	[−0.07, 0.05]	−0.14***	[−0.21, −0.07]
Cong → MH (W_*n*+1_)	−0.05	[−0.19, 0.1]	0	[−0.06, 0.06]	0.03	[−0.07, 0.13]	−0.04	[−0.12, 0.03]	−0.01	[−0.09, 0.07]
SDiv. → Cong → MH (W_*n*_)	0.011	[−0.013, 0.034]	0.003	[−0.013, 0.02]	0.017	[−0.014, 0.048]	−0.001	[−0.013, 0.01]	−0.075**	[−0.127, −0.023]
SDiv. → Cong → MH (W_*n*+1_)	−0.007	[−0.033, 0.019]	0.001	[−0.01, 0.012]	0.015	[−0.039, 0.069]	−0.008	[−0.026, 0.011]	−0.005	[−0.048, 0.039]
Main → Cong	0.03	[−0.28, 0.35]	0.27	[−0.06, 0.59]	0.2	[−0.08, 0.49]	0.27	[−0.06, 0.59]	0.2	[−0.08, 0.49]
Main → Cong → MH (W_*n*_)	0.002	[−0.02, 0.024]	0.005	[−0.018, 0.028]	0.006	[−0.008, 0.021]	−0.002	[−0.018, 0.014]	−0.028	[−0.069, 0.014]
Main → Cong → MH (W_*n*+1_)	−0.001	[−0.016, 0.013]	0.001	[−0.015, 0.017]	0.005	[−0.016, 0.027]	−0.011	[−0.037, 0.014]	−0.002	[−0.018, 0.015]
(C) Only‐youth vs. Congruent (Teacher and youth)										
SDiv. → Cong	0.36*	[0.01, 0.72]	0.6***	[0.35, 0.86]			0.6***	[0.35, 0.86]		
Cong → MH (W_*n*_)	0.03	[−0.01, 0.07]	0.1**	[0.03, 0.16]			−0.03	[−0.08, 0.02]		
Cong → MH (W_*n*+1_)	0.15**	[0.06, 0.25]	0	[−0.05, 0.04]			0.02	[−0.04, 0.08]		
SDiv. → Cong → MH (W_*n*_)	0.01	[−0.008, 0.029]	0.057*	[0.009, 0.105]			−0.018	[−0.046, 0.011]		
SDiv. → Cong → MH (W_*n*+1_)	0.056	[−0.008, 0.12]	−0.003	[−0.03, 0.025]			0.011	[−0.023, 0.046]		
Main → Cong	0.11	[−0.25, 0.48]	−0.03	[−0.32, 0.25]			−0.03	[−0.32, 0.25]		
Main → Cong → MH (W_*n*_)	0.003	[−0.009, 0.015]	−0.003	[−0.03, 0.024]			0.001	[−0.008, 0.01]		
Main → Cong → MH (W_*n*+1_)	0.017	[−0.04, 0.075]	0	[−0.002, 0.002]			−0.001	[−0.006, 0.005]		

*Note*: In this table, the direct effect estimates are presented as standardized coefficients (*β*), while the indirect effect estimates are reported as unstandardized coefficients (*b*).

Abbreviations: CI, confidence interval; Cong, congruent victim; Main, mainly heterosexual; MH, mental health outcomes; SDiv, sexually diverse.

* *p* < .05.

** *p* < .01.

*** *p* < .001.

When comparing the youth and parents congruent class with the only‐parent class (Table 5B), *sexually diverse* youth were again more likely to be in the congruent class, though this was only evident at age 14. This congruence was associated with lower self‐esteem concurrently at that age, but not higher levels of emotional problems. No longitudinal associations were found.

Comparisons between the youth and teachers congruent class and the only‐youth class (Table 5C) showed that *sexually diverse* youth were more likely to be in the congruent class at both ages 7 and 11. Congruence identified at ages 7 and 11 was both related to more serious emotional problems at age 11, but no effects emerged for self‐esteem. Only one significant indirect effect was found: Congruence at age 11 was associated with the concurrent higher emotional problems.

In sum, victimized *sexually diverse* youth were particularly likely to be in the congruent class. After controlling for self‐reported frequency, congruently recognized victimization was still associated with worse mental health.

#### Mainly heterosexual youth

For *mainly heterosexual* youth, results pointed to a greater likelihood of being in the only‐youth class rather than the youth and parents congruent class, but no significant indirect effects emerged. Other than this, no subgroup differences were found across the tested cross‐informant patterns.

#### Gender diverse youth

Comparisons between the youth and parents congruent class and the only‐youth class for gender identity status (Table 6) revealed that *gender diverse* youth were more likely to be in the congruent class from early adolescence onwards (ages 11 and 14). This congruence was associated with worse mental health concurrently. Also, congruence at age 11 was associated with gender diverse youth's worse mental health at follow‐up.

**TABLE 6:** Single informant vs. congruent model with gender identity status.

Only‐youth vs. congruent (parent and youth)										
Paths	Emotional problems						Self‐esteem			
	W4 (Age 7)		W5 (Age 11)		W6 (Age 14)		W5 (Age 11)		W6 (Age 14)	
	*b*/*β*	95% CI	*b*/*β*	95% CI	*b*/*β*	95% CI	*b*/*β*	95% CI	*b*/*β*	95% CI
GD → Cong	0.38	[−0.22, 0.98]	0.65*	[0.16, 1.14]	0.69**	[0.25, 1.13]	0.65*	[0.16, 1.14]	0.69**	[0.25, 1.13]
Cong → MH (Wn)	0.02*	[0, 0.04]	0.09***	[0.05, 0.13]	0.05**	[0.02, 0.08]	−0.06***	[−0.09, −0.04]	−0.08***	[−0.12, −0.05]
Cong → MH (W_*n*+1_)	0.11***	[0.06, 0.16]	0.05**	[0.02, 0.08]	0.03	[−0.02, 0.08]	−0.06***	[−0.1, −0.03]	−0.02	[−0.06, 0.02]
GD → Cong → MH (W_*n*_)	0.008	[−0.007, 0.024]	0.06*	[0.008, 0.113]	0.035*	[0.004, 0.066]	−0.041*	[−0.077, −0.005]	−0.057*	[−0.102, −0.012]
GD → Cong → MH (W_*n*+1_)	0.041	[−0.027, 0.109]	0.03*	[0.001, 0.06]	0.021	[−0.016, 0.058]	−0.041*	[−0.079, −0.002]	−0.015	[−0.044, 0.014]

*Note*: In this table, the direct effect estimates are presented as standardized coefficients (*β*), while the indirect effect estimates are reported as unstandardized coefficients (*b*).

Abbreviations: CI, confidence interval; Cong, congruent victim; GD, gender diverse; MH, mental health outcomes.

* *p* < .05.

** *p* < .01.

*** *p* < .001.

## DISCUSSION

Despite a series of political progress in SGD rights in the United Kingdom since the start of the 21st century (Stonewall, n.d.), victimization disparities, accompanied by detrimental health, persist for British SGD youth (Huang et al., 2024). Victimization disparities between SGD and HC youth reflect social stigma and bias against SGD identities. This requires urgent attention, as identity bias yields threats to one's core values. Knowledge is therefore needed to dismantle the process through which bias is translated into youth's health outcomes. Bias and stigma contribute to both the occurrence and the different recognition patterns of victimization. Whether victimization is recognized by youth, adults (parents and teachers), or both has important implications for youth's mental health. With large national cohort data from the United Kingdom, this preregistered study examined the extent to which the way victimization is recognized would be associated with concurrent and follow‐up mental health disparities for SGD youth beyond victimization frequency, across a long developmental span from middle childhood (age 7) to late adolescence (age 17). Among the three SGD statuses (sexually diverse, mainly heterosexual, and gender diverse) tested, sexually diverse and gender diverse youth demonstrated similar patterns: from middle childhood to middle adolescence (age 14), they were more likely than heterosexual, cisgender youth to be perceived as victims by both themselves and parents/teachers. In early adolescence (age 11), sexually diverse youth specifically were more likely to be seen as victims only by parents. Both patterns were related to greater emotional problems and lower self‐esteem. These results indicate that mental health disparities for sexually diverse and gender diverse youth are not only driven by more frequent victimization, but also by victimization that is visible to parents and teachers. In addition to these disparities, mainly heterosexual youth emerged as an under‐recognized group at risk: From early adolescence, they were more likely to report victimization unacknowledged by parents or teachers, which contributed to mental health disparities for this group. Importantly, all differences remained after controlling for youth's self‐reported victimization frequency. Taken together, these findings extend the minority stress framework by showing that disparities can arise not only from more frequent victimization but also from how victimization is recognized. Based on a national cohort study, they provide crucial information on how recognition relates to heterogeneity in mental health outcomes, which can inform broader theories and serve as a basis for future cross‐national comparison.

### Congruent recognition of victimization among SGD youth

Although parents and teachers often underestimate youth's victimization (Demaray et al., 2013; Volk et al., 2017), we found that peer victimization was particularly more likely to be congruently recognized by both youth and parents/teachers among SGD youth (compared to HC youth). Such recognition disparities emerged already in childhood and were consistently associated with mental health disparities for SGD youth. Several possible mechanisms might explain these findings.

First, parents' and teachers' sensitivity to youth's victimization is related to the vulnerabilities they perceive youth to have (Kretschmer et al., 2025; Stives et al., 2021), which may be directly or indirectly linked to youth's SGD status. Some youth might already experience SGD milestones at a young age (Baams et al., 2025; Bishop et al., 2023). With growing societal awareness of the vulnerability of SGD youth (Russell & Fish, 2019), parents and teachers may be more *vigilant* or attuned to potential stigma toward these children. SGD youth in the current sample grew up with significant political changes in the United Kingdom (Stonewall, n.d.), accompanied by increased media discussion. Inclusive voices arose among the public discussion, yet SGD identities, especially gender diverse identities, were disproportionately presented as stigmatized and victimized (Baker, 2019; Buckle, 2018). This social trend potentially shaped parents' and teachers' awareness and vigilance. This vigilance may not even depend on “outness”: Even when youth do not openly express their romantic feelings or gender identity, they may still be perceived as “different” from the majority by parents and teachers (Bochicchio et al., 2019; Schlehofer & Cortez‐Regan, 2022). This is particularly relevant at younger ages when most youth are not yet certain or open about their identities, yet may exhibit gender‐diverse interests or expressions (Mustanski et al., 2014; Russell & Fish, 2016).

Youth who identify as SGD at a later age may not yet experience or express diverse attractions or identities. Yet, they might experience psychosocial vulnerabilities at a younger age, accompanied by risks of victimization (Huang et al., 2024), due to being different from the majority social norm. Some parents may observe such vulnerabilities or victimization experiences in their child (Benatov, 2019; Kretschmer et al., 2025; Stives et al., 2021), and, similarly, teachers may perceive vulnerabilities in youth who are different from others (Mishna et al., 2006). Taken together, parents' and teachers' vigilance may stem from youth's SGD expression or their early psychosocial vulnerabilities. As this study did not explicitly test how adults learned of victimization, future research should examine the pathways through which adults become aware, and the role of vigilance in shaping congruence reports.

A second possible explanation is that congruent recognition may reflect greater *severity* of victimization among SGD youth, which not only explains parents' and teachers' awareness among SGD youth, but also the correlated worse outcomes. Indeed, we found that congruent recognition was linked to youth's worse mental health beyond a higher frequency of victimization. Minority stress literature shows that such victimization often persists across ages and contexts within the same individuals (Huang et al., 2024; Kaufman et al., 2020; Myers et al., 2020). The intensity could also be characterized by multiple perpetrators and more severe forms (Flores, 2019; Jones et al., 2018; Truman et al., 2024). Such persistence and intensity likely increase the probability that multiple informants, both youth and parents/teachers, observe and report these experiences (De Los Reyes & Ohannessian, 2016; Tremblay‐Perreault et al., 2022). This perspective suggests that parents' and teachers' reports may capture dimensions of severity that are not fully reflected in youth self‐reports of frequency, such as chronicity, pervasiveness, or social visibility. When youth and adult perceptions converge, it may indicate that victimization is both salient and enduring enough to be noticed across perspectives. Given that our measures did not cover other dimensions that define the severity of victimization, future research should adopt a more comprehensive measure of victimization to examine whether informant congruence functions as an indicator of severity, and whether this helps explain disparities in mental health outcomes among SGD youth.

One other possible explanation for why congruently recognized victimization was linked to worse youth outcomes could be parents' and teachers' reactions following awareness. In theory, recognition could enable timely intervention, yet our findings suggest that knowledge alone may fail to protect SGD youth, allowing both victimization and distress to persist. Minority stress theory highlights that stigma at the levels of parents, teachers, and peers can undermine the availability and quality of interventions (Brooks, 1981; Meyer, 1995, 2003). Empirical evidence shows that SGD youth more frequently report unresolved victimization despite adult awareness, and express less trust in parents' or teachers' willingness or ability to help (Kaufman & Baams, 2022). Possible explanations include stigmatizing attitudes among some parents or teachers (Hall & Rodgers, 2019; Robinson, 2018; Ryan et al., 2009), as well as a lack of confidence, knowledge, or perceived efficacy even among those motivated to act (Ioverno et al., 2024; Nappa et al., 2018; Poteat et al., 2011; Swanson & Gettinger, 2016). Stigma‐based rejection is often less malleable than rejection based on non‐stigmatized characteristics, making it harder to address (Link & Phelan, 2001; Pescosolido & Martin, 2015; Phelan et al., 2014). Adults' reactions may not always be helpful (Bjereld et al., 2021), and recognition without effective follow‐up may even reinforce feelings of helplessness and marginalization (Tholander et al., 2020), ultimately exacerbating rather than buffering psychological distress. On the bright side, although congruently recognized victimization can serve as a sign of severity, recognition can be a precondition for further recovery and intervention. To better support SGD youth to shake off the burden of bias, we should equip parents and teachers with a more inclusive way of responding and a more supportive strategy to intervene. It is crucial for future studies to examine whether and how parents and teachers act on their knowledge at different developmental stages, and whether SGD youth perceive such responses as validating, unhelpful, or intrusive.

### Incongruent victimization reports: distinct risks for sexually diverse and mainly heterosexual youth

Beyond congruent recognition, we also found that peer victimization not perceived by adults or unacknowledged by youth themselves was linked to worse outcomes for SGD youth, both concurrently and over time. In early adolescence, sexually diverse youth were more likely than exclusively heterosexual youth to be perceived as victims only by their parents and not by themselves. This pattern possibly reflects a tendency among some sexually diverse youth to downplay or normalize victimization experiences, which can hinder their ability to recognize incidents and create barriers to seeking support (De Heer et al., 2024). As a result, such victimization may be reported only by adults, and this under‐identification by youth may contribute to disparities in mental health outcomes (Goodman, 2013; Tremblay‐Perreault et al., 2022). In situations where parents are concerned but youth do not recognize victimization themselves, it is important to prevent excessive vigilance from adults while also ensuring that youth do not normalize these experiences. The group of gender diverse youth whose victimization was only recognized by parents was very small (*n* = 1 ~ 5), but, given the small sample size, we cannot yield robust results for this group. Future studies should use targeted sampling to examine victimization report patterns among gender diverse youth.

Interestingly, from early adolescence onwards, mainly heterosexual youth were more likely than exclusively heterosexual youth to report victimization unrecognized by adults, which in turn was associated with worse mental health. Mainly heterosexual youth represent a special category on the sexual orientation spectrum: Compared to bisexual individuals, they are less likely to have same‐sex relationships and experiences, and display better adjustment (Savin‐Williams & Vrangalova, 2013; Vrangalova & Savin‐Williams, 2014). Mainly heterosexual individuals typically show more internalized homonegativity and less openness about their sexual orientation than other sexually diverse youth (Kuyper et al., 2016). Our findings highlighted that when parents or teachers failed to recognize their victimized experiences, mainly heterosexual youth experienced more mental distress compared to exclusively heterosexual youth. This suggests a unique form of minority stress, in which more concealed identities and experiences may be problematic. Future research should treat mainly heterosexual youth as a distinct sexual orientation subgroup and investigate the mechanisms through which their peer victimization becomes less visible to parents and teachers: For instance, whether identity concealment is related to the perception, interpretation, and disclosure of victimization.

### Parent reports: greater sensitivity and stronger long‐term relevance

Across both parent–youth and teacher–youth reports, worse mental health among sexually diverse youth was associated with congruently recognized victimization, whereas among mainly heterosexual youth it was linked to youth‐only reported victimization. Nevertheless, several key differences emerged between parent–youth and teacher–youth report patterns. First, in early adolescence, compared to exclusively heterosexual youth, parents were more likely than youth to recognize sexually diverse youth's victimization, but similar patterns were not observed in teacher–youth reports. Parents may be more sensitive to subtle signs of SGD youth's victimization because of their close and protective role. In contrast, teachers may be less informed about incidents that youth are reluctant to disclose at school, particularly when disclosure could involve social risks such as being perceived as snitching or experiencing retaliation (Mishna et al., 2006; Shaw et al., 2019). Teachers may also lack sufficient training to recognize SGD‐related bullying. A report showed that in British schools, the proportion of trained teachers has doubled from 2009 to 2014, yet about 80% of teachers remained untrained (Guasp et al., 2014).

Second, victimization recognized only by parents was more consistently linked to youth's worse mental health, but similar associations among teacher–youth reports were sporadic. Third, while congruent parent–youth recognition was consistently associated with sexually diverse youth's worse mental health longitudinally, congruent teacher–youth recognition was only linked to concurrent outcomes. These results suggested that, compared to teacher reports, parent reports may act as a more robust indicator for youth's mental health, especially longitudinally. Similarly, parent‐reported victimization was identified as a robust predictive factor of youths' longer‐term maladjustment in general youth samples (Kretschmer et al., 2025). Although directly comparing parent–youth and teacher–youth discrepancies was beyond the scope and power of the current study, future research would benefit from integrating parent and teacher reports into a single model.

### Strengths, limitations, and future directions

This study has several notable strengths. It used longitudinal data spanning multiple stages of development, from middle childhood through adolescence, and employed a multi‐informant design that went beyond victimization frequency to include differences in recognition across youth, parents, and teachers. The MCS project, as a large cohort sample in the United Kingdom, enabled comparisons between informants, tracing back SGD‐based disparities into childhood and adolescence. In addition, sexual orientation was measured in a way that distinguished exclusively heterosexual from mainly heterosexual youth, enabling more nuanced analyses of subgroups often overlooked in victimization research (Savin‐Williams & Vrangalova, 2013). Moreover, through the lens of recognition, this study addressed victimization disparities between SGD and HC youth not as individual experiences, but embedded in youth's relational systems. Our findings revealed that identity‐based victimization disparities should be tackled in their social ecology. We encourage future studies to examine the generalizability of the findings across contexts that vary in norms and policies.

However, several measurement issues should be considered when interpreting the findings. First, in the victimization measures, no timeframe was provided for youth's reports, while frequency was not addressed in adults' reports. Although items referring to present experiences without a timeframe were used in other validated bullying scales and may help prevent recall bias in young people (Harbin et al., 2019; Rosen et al., 2013), asking participants to provide the frequency of experiences in a specific timeframe would enhance the validity of the measures (Hall, 2016). Second, the wording of victimization items, although all reflecting general bullying, differed mildly between informants and across time. Thus, items were dichotomized to explore whether victimization was *congruently* recognized across informants. More rigorous comparisons would benefit from using the same wording across informants and time points, comparing cross‐informant *consistency* on the original item level. Third, only one item per informant was used to measure victimization. Although for traditional victimization, a single‐item approach was comparable to a multi‐item scale when comparing groups (Yanagida et al., 2016), single‐item approaches may still underestimate the overall prevalence rate and variability of victimization (Jetelina et al., 2019). Altogether, while these measurement limitations are not likely to affect our findings regarding between‐group disparities for SGD and HC youth, they may involve systematic bias.

Another limitation concerns the measurement and coding of SGD status once and only at late adolescence (W7). Although this is a common research strategy in this field, it underrepresents the SGD population in two aspects. First, the fluidity of sexual orientation and gender identity across development could not be captured. Second, although most SGD youth encounter milestones in adolescence, a small proportion of them may not yet have these experiences until adulthood (Bishop et al., 2023; Puckett et al., 2022). We encourage future studies to include earlier measures of SGD status, and to track SGD status prospectively into adulthood. It would be valuable to see how the development of SGD‐related identities relates to the emergence of SGD‐related psychosocial disparities.

This study also has several other limitations. First, the reliability of W4 emotional problems was low. The findings using this measure should be interpreted with caution. Second, although the longitudinal analyses supported associations over time, causality and directionality cannot be inferred. Future studies should adopt longitudinal designs to examine reverse or reciprocal effects among mental health and victimization, as well as changes in cross‐informant patterns over time (Nylund‐Gibson et al., 2014; Usami, 2021). Furthermore, while the current study focused on SGD youth, future research should assess the generalizability of these findings to other marginalized groups (Jackson et al., 2016).

Also, our findings should be interpreted in light of the British social context at the time the data were collected (2008–2018). During this period, the United Kingdom was considered a leader in SGD rights. However, acceptance of sexual and gender diversity has changed over time and varies substantially across countries. Since 2019, the United Kingdom's ranking on the inclusion index dropped sharply, falling below the European average by 2026 (ILGA‐Europe, 2026). Although sexual diversity remained socially accepted and legally recognized, policies and public attitudes toward gender diversity deteriorated; For instance, the Gender Recognition Reform Bill was blocked by the United Kingdom Government in 2023 (Stonewall, n.d.; Clery, 2023). Future studies may examine whether societal changes and differences in social and legal contexts across countries influence disparities for gender‐diverse youth.

## CONCLUSION

This study showed that disparities among SGD youth are not limited to a higher frequency of victimization, and that single‐informant research risks underestimating victimization disparities and the correlated health disparities. Our findings underscore the importance of enhancing adults' awareness, both of victimization and of *how* to intervene effectively. Although adults generally underestimate youths' peer victimization, peer victimization among SGD youth (compared to HC youth) is more likely to be congruently recognized by both youth and adults, often from a young age. Such visibility, while it could be an intervention opportunity, was consistently associated with poorer outcomes, indicating that knowledge alone is insufficient. Our results also highlighted nuances in the disparities of mainly heterosexual youth compared to exclusively heterosexual or other sexually diverse youth, showing the importance of differentiating these groups in research on victimization and mental health disparities. Victimization recognition disparities were evident as early as middle childhood, underscoring the urgency of equipping parents and teachers with the skills to translate awareness into effective support. Future research should therefore focus on when and how adults should act upon their knowledge, to identify age‐appropriate strategies that ensure recognition leads to real change. By addressing the gap between awareness and effective intervention, and by attending to the distinct risks observed for mainly heterosexual youth, we can work toward reducing the persistent mental health disparities faced by SGD groups.

## AUTHOR CONTRIBUTIONS

**Jingyi Huang:** Conceptualization; writing – original draft; data curation; formal analysis; writing – review and editing. **Tessa M. L. Kaufman:** Conceptualization; formal analysis; supervision; writing – original draft; writing – review and editing. **Susan Branje:** Conceptualization; formal analysis; supervision; writing – review and editing.

## FUNDING INFORMATION

No funding was received for this research.

## CONFLICT OF INTEREST STATEMENT

The authors declare that they have no conflict of interest.

## ETHICAL APPROVAL

Regarding data collection, the Millennium Cohort Study (MCS) obtained its ethical approval through the National Health Service (NHS) Research Ethics Committee (REC) system in the United Kingdom. A series of approvals was reached for each wave's data collection (MREC/01/6/19, MREC/03/2/022, 05/MRE02/46, 07/MRE03/32, 11/YH/ 0203, 13/LO/1786, and 17/NE/0341 for wave 1 to wave 7, respectively). The current study obtained ethical approval for analyzing the data through the Ethics Committee of the Faculty of Social and Behavioral Sciences of Utrecht University (file number 25‐0368).

## INFORMED CONSENT

Informed consent was obtained from all individual participants included in the study.

## DECLARATION OF GENERATIVE AI AND AI‐ASSISTED TECHNOLOGIES IN THE WRITING PROCESS

During the preparation of this work (July 2024 to Sep 2026), the authors used ChatGPT (model version not visible to free users) and Claude (Opus 4.6) to check for grammar mistakes and to ensure accurate English wording. Data and model outputs were not uploaded to AI. Before using AI for proofreading, the authors drafted the manuscript in the first place. After using AI, the authors reviewed and edited the content as needed and took full responsibility for the content of the publication.

## Supporting information


Appendix S1.–S7.

## Data Availability

The data that support the findings of this study are available from UK Data Service. Restrictions apply to the availability of these data, which were used under license for this study. Data are available from https://ukdataservice.ac.uk/ with the permission of UK Data Service. Materials and Code Availability: The code and output files for the analysis are available at https://osf.io/ekabu/?view_only=8ebdb18a4b4e41e5b80f6ae94e349ac2.

## References

[jora70283-bib-0001] Amos, R. , Manalastas, E. J. , White, R. , Bos, H. , & Patalay, P. (2020). Mental health, social adversity, and health‐related outcomes in sexual minority adolescents: A contemporary national cohort study. The Lancet Child & Adolescent Health, 4(1), 36–45. 10.1016/S2352-4642(19)30339-6 31753807

[jora70283-bib-0002] Angold, A. , Costello, E. J. , Messer, S. C. , & Pickles, A. (1995). Development of a short questionnaire for use in epidemiological studies of depression in children and adolescents. International Journal of Methods in Psychiatric Research, 5(4), 237–249.

[jora70283-bib-0003] Baams, L. , Caprinali, A. , & Fischer, M. (2025). A systematic review of the timing of sexual and gender identity milestones. In L. Baams & T. M. L. Kaufman (Eds.), Sexually and gender diverse adolescents: Critical perspectives on risk and resilience. Routledge.

[jora70283-bib-0004] Baams, L. , & Kaufman, T. M. L. (2023). Sexual orientation and gender identity/expression in adolescent research: Two decades in review. The Journal of Sex Research, 60(7), 1004–1019. 10.1080/00224499.2023.2219245 37307300

[jora70283-bib-0005] Baker, P. (2019). Comparison of the coverage of stories about trans people in 2012 and 2018–19 . https://mermaidsuk.org.uk/news/exclusive‐mermaids‐research‐into‐newspaper‐coverage‐on‐trans‐issues/

[jora70283-bib-0006] Bayram Özdemir, S. , Caravita, S. C. S. , & Thornberg, R. (2024). Bias‐based harassment and bullying: Addressing mechanisms and outcomes for possible interventions. European Journal of Developmental Psychology, 21(4), 505–519. 10.1080/17405629.2024.2376047

[jora70283-bib-0007] Benatov, J. (2019). Parents' feelings, coping strategies, and sense of parental self‐efficacy when dealing with children's victimization experiences. Frontiers in Psychiatry, 10, 700. 10.3389/fpsyt.2019.00700 PMC6788459 31636575

[jora70283-bib-0008] Bishop, M. D. , Mallory, A. B. , & Russell, S. T. (2023). Sexual minority identity development: Latent profiles of developmental milestones in a national probability sample. Psychology of Sexual Orientation and Gender Diversity, 10(4), 622–637. 10.1037/sgd0000569 PMC10756425 38162689

[jora70283-bib-0009] Bjereld, Y. , Daneback, K. , & Mishna, F. (2021). Adults' responses to bullying: The victimized youth's perspectives. Research Papers in Education, 36(3), 257–274. 10.1080/02671522.2019.1646793

[jora70283-bib-0010] Bochicchio, V. , Perillo, P. , Valenti, A. , Chello, F. , Amodeo, A. L. , Valerio, P. , & Scandurra, C. (2019). Pre‐service teachers' approaches to gender‐nonconforming children in preschool and primary school: Clinical and educational implications. Journal of Gay & Lesbian Mental Health, 23(2), 117–144. 10.1080/19359705.2019.1565791

[jora70283-bib-0011] Bockting, W. O. (2014). Transgender identity development. In D. L. Tolman , L. M. Diamond , J. A. Bauermeister , W. H. George , J. G. Pfaus , & L. M. Ward (Eds.), APA handbook of sexuality and psychology, vol. 1: Person‐based approaches (pp. 739–758). American Psychological Association. 10.1037/14193-024

[jora70283-bib-0012] Brooks, V. (1981). Minority stress and lesbian women. Lexington Books.

[jora70283-bib-0013] Buckle, S. (2018). Homosexuality on the small screen: Television and gay identity in Britain. Bloomsbury Publishing.

[jora70283-bib-0014] Clery, E. (2023). A liberalisation in attitudes? National Centre for Social Research. https://natcen.ac.uk/publications/bsa‐40‐liberalisation‐attitudes

[jora70283-bib-0015] Connelly, R. , & Platt, L. (2014). Cohort profile: UK millennium cohort study (MCS). International Journal of Epidemiology, 43(6), 1719–1725. 10.1093/ije/dyu001 24550246

[jora70283-bib-0016] Copen, C. E. , Chandra, A. , & Febo‐Vazquez, I. (2016). *Sexual behavior, sexual attraction, and sexual orientation among adults aged 18–44 in the United States: Data from the 2011–2003 National Survey of Family Growth* (Vol. 88). https://stacks.cdc.gov 26766410

[jora70283-bib-0017] De Heer, B. A. , Lipschutz, S. , & Shevat, S. (2024). “I was just so confused, like does this even count as sexual assault?”: Understanding LGBTQA+ sexual victimization, help‐seeking, and mental health outcomes. Journal of Gender Studies, 33(7), 943–957. 10.1080/09589236.2023.2260323 PMC11665924 39720815

[jora70283-bib-0018] De Los Reyes, A. , & Ohannessian, C. M. (2016). Introduction to the special issue: Discrepancies in adolescent–parent perceptions of the family and adolescent adjustment. Journal of Youth and Adolescence, 45(10), 1957–1972. 10.1007/s10964-016-0533-z 27384957

[jora70283-bib-0019] Demaray, M. K. , Malecki, C. K. , Secord, S. M. , & Lyell, K. M. (2013). Agreement among students', teachers', and parents' perceptions of victimization by bullying. Children and Youth Services Review, 35(12), 2091–2100. 10.1016/j.childyouth.2013.10.018

[jora70283-bib-0020] Diamond, L. M. , & Alley, J. (2022). Rethinking minority stress: A social safety perspective on the health effects of stigma in sexually‐diverse and gender‐diverse populations. Neuroscience & Biobehavioral Reviews, 138, 104720. 10.1016/j.neubiorev.2022.104720 35662651

[jora70283-bib-0021] Flores, A. R. (2019). Social Acceptance of LGBT People in 174 Countries: 1981 to 2017 . https://escholarship.org/content/qt5qs218xd/qt5qs218xd.pdf

[jora70283-bib-0022] Goodman, A. , & Goodman, R. (2011). Population mean scores predict child mental disorder rates: Validating SDQ prevalence estimators in Britain. Journal of Child Psychology and Psychiatry, 52(1), 100–108. 10.1111/j.1469-7610.2010.02278.x 20722879

[jora70283-bib-0023] Goodman, K. (2013). Parent–youth discrepancies in ratings of youth victimization: Associations with psychological adjustment. American Journal of Orthopsychiatry, 83(1), 37–46. 10.1111/ajop.12010 23330621

[jora70283-bib-0024] Goodman, K. L. , De Los Reyes, A. , & Bradshaw, C. P. (2010). Understanding and using informants' reporting discrepancies of youth victimization: A conceptual model and recommendations for research. Clinical Child and Family Psychology Review, 13(4), 366–383. 10.1007/s10567-010-0076-x 20799062

[jora70283-bib-0025] Guasp, A. , Ellison, G. , & Satara, T. (2014). The Teachers' Report . Stonewall. https://www.stonewall.org.uk/resources/teachers‐report‐2014

[jora70283-bib-0026] Hall, W. J. (2016). Initial development and validation of the Bullyharm: The bullying, harassment, and aggression receipt measure. Psychology in the Schools, 53(9), 984–1000. 10.1002/pits.21957 PMC5303007 28194041

[jora70283-bib-0027] Hall, W. J. , & Rodgers, G. K. (2019). Teachers' attitudes toward homosexuality and the lesbian, gay, bisexual, and queer community in the United States. Social Psychology of Education, 22(1), 23–41. 10.1007/s11218-018-9463-9

[jora70283-bib-0028] Harbin, S. M. , Kelley, M. L. , Piscitello, J. , & Walker, S. J. (2019). Multidimensional bullying victimization scale: Development and validation. Journal of School Violence, 18(1), 146–161. 10.1080/15388220.2017.1423491

[jora70283-bib-0029] Huang, J. , Kaufman, T. M. L. , Baams, L. , & Branje, S. (2024). Peer bullying victimization trajectories for sexually and gender diverse youth from early childhood to late adolescence. Journal of Youth and Adolescence, 53(11), 2589–2609. 10.1007/s10964-024-02020-8 PMC11466994 38849685

[jora70283-bib-0030] ILGA‐Europe . (2026). Rainbow Map . https://rainbowmap.ilga‐europe.org/

[jora70283-bib-0031] Ioverno, S. , Nappa, M. R. , McCurdy, A. , James, I. , Pistella, J. , Baiocco, R. , & Russell, S. T. (2024). Empowering teacher intention to intervene: The role of self‐efficacy in addressing homophobic bullying. School Mental Health, 16(4), 1293–1310. 10.1007/s12310-024-09694-2

[jora70283-bib-0032] Jackson, J. W. , Williams, D. R. , & VanderWeele, T. J. (2016). Disparities at the intersection of marginalized groups. Social Psychiatry and Psychiatric Epidemiology, 51(10), 1349–1359. 10.1007/s00127-016-1276-6 PMC5350011 27531592

[jora70283-bib-0033] Jetelina, K. K. , Reingle Gonzalez, J. M. , Cuccaro, P. M. , Peskin, M. F. , Pompeii, L. , Atem, F. , Elliott, M. N. , Earnshaw, V. A. , Davies, S. L. , & Schuster, M. A. (2019). Self‐reporting discrepancies of bullying victimization and perpetration measures. Annals of Epidemiology, 32, 58–63. 10.1016/j.annepidem.2019.01.008 30799205

[jora70283-bib-0034] Jones, L. M. , Mitchell, K. J. , Turner, H. A. , & Ybarra, M. L. (2018). Characteristics of bias‐based harassment incidents reported by a national sample of U.S. adolescents. Journal of Adolescence, 65(1), 50–60. 10.1016/j.adolescence.2018.02.013 29547771

[jora70283-bib-0035] Kaufman, T. M. L. , & Baams, L. (2022). Disparities in perpetrators, locations, and reports of victimization for sexual and gender minority adolescents. Journal of Adolescent Health, 70(1), 99–107. 10.1016/j.jadohealth.2021.06.024 PMC7612809 34446345

[jora70283-bib-0036] Kaufman, T. M. L. , Baams, L. , & Veenstra, R. (2020). Disparities in persistent victimization and associated internalizing symptoms for heterosexual versus sexual minority youth. Journal of Research on Adolescence, 30(S2), 516–531. 10.1111/jora.12495 PMC7064905 30927389

[jora70283-bib-0037] Kessler, R. C. , Andrews, G. , Colpe, L. J. , Hiripi, E. , Mroczek, D. K. , Normand, S.‐L. T. , Walters, E. E. , & Zaslavsky, A. M. (2002). Short screening scales to monitor population prevalences and trends in non‐specific psychological distress. Psychological Medicine, 32(6), 959–976. 10.1017/S0033291702006074 12214795

[jora70283-bib-0038] Kretschmer, T. , van der Ploeg, R. , & Kaufman, T. (2025). Peer victimization in early adolescence and maladjustment in adulthood. European Child & Adolescent Psychiatry, 34(3), 1011–1024. 10.1007/s00787-024-02532-5 PMC11909069 39073497

[jora70283-bib-0039] Kuyper, L. , de Roos, S. , Iedema, J. , & Stevens, G. (2016). Growing up with the right to marry: Sexual attraction, substance use, and well‐being of Dutch adolescents. Journal of Adolescent Health, 59(3), 276–282. 10.1016/j.jadohealth.2016.05.010 27423901

[jora70283-bib-0040] Lambe, L. J. , Farrell, A. H. , & Craig, W. (2025). Identity‐based bullying victimization among Canadian adolescents: Experiences of transgender and gender diverse youth. Journal of Interpersonal Violence, Advanced online publication. 10.1177/08862605251363627 PMC13669462 40879214

[jora70283-bib-0041] Link, B. G. , & Phelan, J. C. (2001). Conceptualizing stigma. Annual Review of Sociology, 27, 363–385. 10.1146/annurev.soc.27.1.363

[jora70283-bib-0042] Martin‐Storey, A. , & Fish, J. (2019). Victimization disparities between heterosexual and sexual minority youth from ages 9 to 15. Child Development, 90(1), 71–81. 10.1111/cdev.13107 PMC6309771 29938786

[jora70283-bib-0043] Martos, A. J. , Nezhad, S. , & Meyer, I. H. (2015). Variations in sexual identity milestones among lesbians, gay men, and bisexuals. Sexuality Research & Social Policy, 12(1), 24–33. 10.1007/s13178-014-0167-4 PMC5042327 27695579

[jora70283-bib-0044] Meyer, I. H. (1995). Minority stress and mental health in gay men. Journal of Health and Social Behavior, 36(1), 38–56. 10.2307/2137286 7738327

[jora70283-bib-0045] Meyer, I. H. (2003). Prejudice, social stress, and mental health in lesbian, gay, and bisexual populations: Conceptual issues and research evidence. Psychological Bulletin, 129(5), 674–697. 10.1037/0033-2909.129.5.674 PMC2072932 12956539

[jora70283-bib-0046] Mishna, F. , Pepler, D. , & Wiener, J. (2006). Factors associated with perceptions and responses to bullying situations by children, parents, teachers, and principals. Victims & Offenders, 1(3), 255–288. 10.1080/15564880600626163

[jora70283-bib-0047] Mustanski, B. , Kuper, L. , & Greene, G. J. (2014). Development of sexual orientation and identity. In D. L. Tolman , L. M. Diamond , J. A. Bauermeister , W. H. George , J. G. Pfaus , & L. M. Ward (Eds.), APA handbook of sexuality and psychology, vol. 1: Person‐based approaches (pp. 597–628). American Psychological Association. 10.1037/14193-019

[jora70283-bib-0048] Myers, W. , Turanovic, J. J. , Lloyd, K. M. , & Pratt, T. C. (2020). The victimization of LGBTQ students at school: A meta‐analysis. Journal of School Violence, 19(4), 421–432. 10.1080/15388220.2020.1725530

[jora70283-bib-0049] Nappa, M. R. , Palladino, B. E. , Menesini, E. , & Baiocco, R. (2018). Teachers' reaction in homophobic bullying incidents: The role of self‐efficacy and homophobic attitudes. Sexuality Research & Social Policy, 15(2), 208–218. 10.1007/s13178-017-0306-9

[jora70283-bib-0050] Nylund‐Gibson, K. , Grimm, R. , Quirk, M. , & Furlong, M. (2014). A latent transition mixture model using the three‐step specification. Structural Equation Modeling: A Multidisciplinary Journal, 21(3), 439–454. 10.1080/10705511.2014.915375

[jora70283-bib-0051] Pescosolido, B. A. , & Martin, J. K. (2015). The stigma complex. Annual Review of Sociology, 41, 87–116. 10.1146/annurev-soc-071312-145702 PMC4737963 26855471

[jora70283-bib-0052] Phelan, J. C. , Lucas, J. W. , Ridgeway, C. L. , & Taylor, C. J. (2014). Stigma, status, and population health. Social Science & Medicine, Structural Stigma and Population Health, 103, 15–23. 10.1016/j.socscimed.2013.10.004 PMC4091623 24507907

[jora70283-bib-0053] Poteat, V. P. , Mereish, E. H. , DiGiovanni, C. D. , & Koenig, B. W. (2011). The effects of general and homophobic victimization on adolescents' psychosocial and educational concerns: The importance of intersecting identities and parent support. Journal of Counseling Psychology, 58(4), 597–609. 10.1037/a0025095 21859187

[jora70283-bib-0054] Puckett, J. A. , Tornello, S. , Mustanski, B. , & Newcomb, M. E. (2022). Gender variations, generational effects, and mental health of transgender people in relation to timing and status of gender identity milestones. Psychology of Sexual Orientation and Gender Diversity, 9(2), 165–178. 10.1037/sgd0000391 PMC9380989 35983565

[jora70283-bib-0055] Robinson, B. A. (2018). Conditional families and lesbian, gay, bisexual, transgender, and queer youth homelessness: Gender, sexuality, family instability, and rejection. Journal of Marriage and Family, 80(2), 383–396. 10.1111/jomf.12466

[jora70283-bib-0056] Rosen, L. H. , Beron, K. J. , & Underwood, M. K. (2013). Assessing peer victimization across adolescence: Measurement invariance and developmental change. Psychological Assessment, 25(1), 1–11. 10.1037/a0028985 PMC3736851 22708574

[jora70283-bib-0057] Rosenberg, M. (1989). Society and the adolescent self‐image. Wesleyan University Press.

[jora70283-bib-0058] Russell, S. T. , & Fish, J. N. (2016). Mental health in lesbian, gay, bisexual, and transgender (LGBT) youth. Annual Review of Clinical Psychology, 12(1), 465–487. 10.1146/annurev-clinpsy-021815-093153 PMC4887282 26772206

[jora70283-bib-0059] Russell, S. T. , & Fish, J. N. (2019). Sexual minority youth, social change, and health: A developmental collision. Research in Human Development, 16(1), 5–20. 10.1080/15427609.2018.1537772 PMC6786797 31602178

[jora70283-bib-0060] Ryan, C. , Huebner, D. , Diaz, R. M. , & Sanchez, J. (2009). Family rejection as a predictor of negative health outcomes in White and Latino lesbian, gay, and bisexual young adults. Pediatrics, 123(1), 346–352. 10.1542/peds.2007-3524 19117902

[jora70283-bib-0061] Savin‐Williams, R. C. , & Vrangalova, Z. (2013). Mostly heterosexual as a distinct sexual orientation group: A systematic review of the empirical evidence. Developmental Review, 33(1), 58–88. 10.1016/j.dr.2013.01.001

[jora70283-bib-0062] Schacter, H. L. , Marusak, H. A. , Borg, B. A. , & Jovanovic, T. (2024). Facing ambiguity: Social threat sensitivity mediates the association between peer victimization and adolescent anxiety. Development and Psychopathology, 36(1), 112–120. 10.1017/S0954579422001018 PMC10151014 36200351

[jora70283-bib-0063] Schlehofer, M. M. , & Cortez‐Regan, L. (2022). Early reactions of parents to their trans and gender non‐conforming children. LGBTQ+ Family: An Interdisciplinary Journal, 18(1), 81–99. 10.1080/27703371.2021.2023374

[jora70283-bib-0064] Shaw, T. , Campbell, M. A. , Eastham, J. , Runions, K. C. , Salmivalli, C. , & Cross, D. (2019). Telling an adult at school about bullying: Subsequent victimization and internalizing problems. Journal of Child and Family Studies, 28(9), 2594–2605. 10.1007/s10826-019-01507-4

[jora70283-bib-0065] Stives, K. L. , May, D. C. , Mack, M. , & Bethel, C. L. (2021). Understanding responses to bullying from the parent perspective. Frontiers in Education, 6, 1–10. 10.3389/feduc.2021.642367

[jora70283-bib-0066] Stonewall . (2017). School Report . Stonewall. https://www.stonewall.org.uk/resources/school‐report‐2017

[jora70283-bib-0067] Stonewall . (n.d.). Key dates for LGBTQ+ equality . Stonewall UK. https://www.stonewall.org.uk/resources/key‐dates‐lesbian‐gay‐bi‐and‐trans‐equality

[jora70283-bib-0068] Suen, L. W. , Lunn, M. R. , Katuzny, K. , Finn, S. , Duncan, L. , Sevelius, J. , Flentje, A. , Capriotti, M. R. , Lubensky, M. E. , Hunt, C. , Weber, S. , Bibbins‐Domingo, K. , & Obedin‐Maliver, J. (2020). What sexual and gender minority people want researchers to know about sexual orientation and gender identity questions: A qualitative study. Archives of Sexual Behavior, 49(7), 2301–2318. 10.1007/s10508-020-01810-y PMC7497435 32875381

[jora70283-bib-0069] Swanson, K. , & Gettinger, M. (2016). Teachers' knowledge, attitudes, and supportive behaviors toward LGBT students: Relationship to gay‐straight alliances, antibullying policy, and teacher training. Journal of LGBT Youth, 13(4), 326–351. 10.1080/19361653.2016.1185765

[jora70283-bib-0070] Tholander, M. , Lindberg, A. , & Svensson, D. (2020). “A freak that no one can love”: Difficult knowledge in testimonials on school bullying. Research Papers in Education, 35(3), 359–377. 10.1080/02671522.2019.1568534

[jora70283-bib-0071] Thompson, E. M. , & Morgan, E. M. (2008). “Mostly straight” young women: Variations in sexual behavior and identity development. Developmental Psychology, 44(1), 15–21. 10.1037/0012-1649.44.1.15 18194001

[jora70283-bib-0072] Toomey, R. B. (2021). Advancing research on minority stress and resilience in trans children and adolescents in the 21st century. Child Development Perspectives, 15(2), 96–102. 10.1111/cdep.12405

[jora70283-bib-0073] Tremblay‐Perreault, A. , Hébert, M. , & Amédée, L. M. (2022). A latent class analysis of parent–child discrepancies in reports of peer victimization: Associations to child sexual abuse status and psychological adjustment. Development and Psychopathology, 34(3), 889–900. 10.1017/S0954579420001522 33478619

[jora70283-bib-0074] Truman, J. L. , Morgan, R. E. , & Coen, E. J. (2024). Characteristics and consequences of violent victimization in sexual and gender minority communities: An analysis of the 2017–2021 National Crime Victimization Survey. LGBT Health, 11(7), 552–562. 10.1089/lgbt.2023.0110 38757605

[jora70283-bib-0075] Usami, S. (2021). On the differences between general Cross‐lagged panel model and random‐intercept Cross‐lagged panel model: Interpretation of Cross‐lagged parameters and model choice. Structural Equation Modeling: A Multidisciplinary Journal, 28(3), 331–344. 10.1080/10705511.2020.1821690

[jora70283-bib-0076] Volk, A. A. , Veenstra, R. , & Espelage, D. L. (2017). So you want to study bullying? Recommendations to enhance the validity, transparency, and compatibility of bullying research. Aggression and Violent Behavior, 36, 34–43. 10.1016/j.avb.2017.07.003

[jora70283-bib-0077] Vrangalova, Z. , & Savin‐Williams, R. C. (2014). Psychological and physical health of mostly heterosexuals: A systematic review. The Journal of Sex Research, 51(4), 410–445. 10.1080/00224499.2014.883589 24754361

[jora70283-bib-0078] White, J. , Trinh, M.‐H. , & Reynolds, C. A. (2023). Psychological distress, self‐harm and suicide attempts in gender minority compared with cisgender adolescents in the UK. BJPsych Open, 9(5), e138. 10.1192/bjo.2023.534 PMC10486222 37525614

[jora70283-bib-0079] Whittle, K. , Moore, E. , & Stallard, P. (2024). Self‐harm, suicidal ideation, depression and peer relationships in transgender and gender diverse adolescents accessing specialist mental health services. Child and Adolescent Mental Health, 29(4), 363–370. 10.1111/camh.12738 39375974

[jora70283-bib-0080] Wilson, K. (2020). Attitudes toward LGBT people and their rights in Europe. In E. Hannah (Ed.), Oxford research encyclopedia of politics. Oxford University Press. 10.1093/acrefore/9780190228637.013.1335

[jora70283-bib-0081] Yanagida, T. , Gradinger, P. , Strohmeier, D. , Solomontos‐Kountouri, O. , Trip, S. , & Bora, C. (2016). Cross‐national prevalence of traditional bullying, traditional victimization, cyberbullying and cyber‐victimization: Comparing single‐item and multiple‐item approaches of measurement. International Journal of Developmental Science, 10(1–2), 21–32. 10.3233/DEV-150173

