# Supplementary material for: Peer victimization disparities for sexual and gender diverse youth: Cross‐informant reports and associated mental health
Source: J Res Adolesc. 2026 Oct 6;36(4):e70283. doi: 10.1111/jora.70283 (PMC13639502; doi:10.1111/jora.70283)
Supplement: Supplementary file 1 [file JORA-36-0-s001.docx]

**Supplementary Appendix S1. Confirmatory Factor Analysis (CFA)**

| **Table S1** | | | | | |
| --- | --- | --- | --- | --- | --- |
| Confirmatory factor analysis: Emotional Problems | | | | | |
| Wave | Item | Standardized factor loading | | | |
|  |  | All items | Remove item 4 | Remove items 4, 6 | Remove items 4, 6, 5 |
| Wave 4 | W4#1 (happy) | 0.404 | 0.4 | 0.365 | 0.351 |
|  | W4#2 (worry) | 0.364 | 0.367 | 0.385 | 0.382 |
|  | W4#3 (sad) | 0.526 | 0.529 | 0.537 | 0.572 |
|  | W4#4 (quiet) | -0.034 | - | - | - |
|  | W4#5 (alone) | 0.273 | 0.276 | 0.271 | - |
|  | W4#6 (laugh) | 0.181 | 0.18 | - | - |
|  | W4#7 (lost temper) | 0.382 | 0.379 | 0.392 | 0.368 |
| Wave | Item | All items | Remove item 5 |  |  |
| Wave 5 | W5#1 (happy) | 0.347 | 0.336 |  |  |
|  | W5#2 (worry) | 0.635 | 0.64 |  |  |
|  | W5#3 (sad) | 0.734 | 0.731 |  |  |
|  | W5#4 (scared) | 0.687 | 0.691 |  |  |
|  | W5#5 (laugh) | 0.166 | - |  |  |
|  | W5#6 (angry) | 0.571 | 0.572 |  |  |
| Wave | Model | Model fit | | | |
|  |  | RMSEA | CFI | TLI | SRMR |
| Wave 4 | All items | 0.053 | 0.811 | 0.716 | 0.033 |
|  | Remove item 4 | 0.054 | 0.86 | 0.767 | 0.031 |
|  | Remove items 4, 6 | 0.038 | 0.954 | 0.909 | 0.018 |
|  | Remove items 4, 6, 5 | 0.054 | 0.953 | 0.859 | 0.019 |
| Wave 5 | All items | 0.087 | 0.908 | 0.847 | 0.045 |
|  | Remove item 5 | 0.086 | 0.946 | 0.892 | 0.03 |
| Abbreviations: RMSEA, root mean square error of approximation; CFI, comparative fit index; TLI, Tucker-Lewis index; SRMR, standardized root mean square residual. | | | | | |

**Supplementary Appendix S2. Deviation from Pre-registration**

There were three deviations from the pre-registration because of practical reasons, which are explained below in more detail. All deviations were made to enhance the research design and validity of the findings.

First, while we originally proposed using latent class analysis to identify cross-informant victimization patterns, this turned out to be practically infeasible. The planned models relied on two indicators—youth-reported victimization (3-5 point scales) and adult-reported victimization (3-point scales). When both were specified as continuous variables, the models failed to converge due to the limited variance in adult reports relative to youth reports. When adult reports were treated as categorical, the model was not identifiable because two indicators did not provide sufficient degrees of freedom to estimate the thresholds. Given these technical constraints, we instead manually categorized participants into cross-informant victimization classes based on dichotomized victimization reports (0 = not victimized, 1 = victimized), which allowed us to maintain the conceptual framework of our research question while ensuring analytical feasibility.

Second, we changed our approach to longitudinal outcomes. While our pre-registration proposed using Wave 7 (W7, age 17) mental health as the longitudinal outcome, the time interval between late childhood/early adolescent victimization and age 17 outcomes would be excessive for meaningful association. Therefore, we instead used mental health outcomes from the following wave as longitudinal indicators.

Third, we excluded W7 from our cross-informant victimization pattern analysis due to a change in measurement. While youth-reported peer victimization was measured using frequency scales from W4 to W6, the W7 assessment shifted to an agreement scale. This measurement inconsistency would confound the interpretation of developmental changes in victimization patterns across ages.

**Supplementary Appendix S3. Researcher Reflexivity Statement**

The authors of this study are trained in developmental, educational, or clinical psychology, or pedagogical sciences. Two of the authors identified as sexually diverse, cisgender women. One author identified as a cisgender, heterosexual woman. Together, we share a commitment to understanding the complex person-environment interactions that explain youth development and promoting equity and inclusion in youth development, which underlies the motivation for this research.

Victimization is one of the major research interests for the first author, largely driven by previous personal experiences. These experiences became motivation to support victimized youth by better understanding different features of victimization. Also, previous experiences have shaped the sensitivity to how victimization could be normalized, downplayed, or invisible among youth and their close others.

Although this study focused on victimization disparities through a bias-based theoretical framework, we want to emphasize that we do not view sexual and gender diverse (SGD) youth as merely vulnerable. Instead, we are committed to seeing SGD youth as resilient individuals with great potential. We see victimization as more than individual experiences, and we believe that bias-related processes should be understood as embedded in dynamic social contexts. With such a perspective, our aim was not to repeat the negativity of social stigma but to highlight that there are multiple steps along the pathway from bias to health outcomes where we can better support SGD youth’s development.

We acknowledge that interpreting p-values, effect sizes, and model outputs relies on how the researchers frame the meaning of the data. The study was preregistered to minimize bias in the selection of methods and analyses, and in the interpretation of results. The Millennium Cohort Study (MCS) offered a valuable opportunity to trace SGD youth’s development from early ages and examine more subtle intergroup disparities in victimization and health outcomes using a national sample. Using secondary data, we have no relationship with the participants. We chose to combine reports from multiple informants to examine recognition as an entry point into victimization disparities. With this study, we want to open the door to further research with more targeted measures.

**Supplementary Appendix S4. Missingness Analyses**

Based on the selection process (Figure S1), we compared the differences between the included and excluded participants. First, based on the full W7 sample (*n_1_* = 10,952), we compared the 1016 excluded participants with the current analytical sample (*n_4_* = 9936) on covariates (sex assigned at birth, age, and ethnicity), W4-W6 victimization reports, and W7 mental health. Results showed that, compared to the excluded participants, the included participants were more likely to be girls (*χ*²/df = 38.93, *p* < .001), had higher levels of youth-reported peer victimization at W6 (*t* = -2.89, *p* = .004), lower levels of parents-reported peer victimization at W4 (*t* = 2.72, *p* = .007), and higher levels of teachers-reported peer victimization at W4 (*t* = -2.45, *p* = 0.015). All other comparisons were not significant. Second, based on the sample with valid SGD reports (*n_3_* = 10,106), we compared the 170 excluded participants with the analytical sample (*n_4_* = 9936) on SGD status. No significant differences were found. Third, within the analytical sample, pairwise deletion was applied for the parent-youth and teacher-youth model, which excluded participants with victimization reported only by youth or only by parents or teachers. The number of participants included and excluded in pairwise deletion, as well as the mean-level comparison of victimization across included versus excluded participants, are presented in Tables S2-S3. As shown, half of the comparisons showed no significant mean-level differences between excluded and included cases. Among the other half of comparisons with significant differences, the level of victimization was mostly lower among included cases than among excluded cases.

The amount of missing data in the mental health variables ranged from 0.1%-11.8% (Table S4). Little’s Missing Completely At Random (MCAR) test was conducted on all studied variables except for teacher-reported data. The MCS project originally had less teacher data compared to parent or youth data, which makes the missingness of teacher data not comparable. The result was significant (χ²/df = 1.17, p < .001), indicating our data was not MCAR. However, with the χ²/df value below 2 or even 5, the deviation from MCAR was considered acceptable (Alavi et al., 2020; Wheaton et al., 1977). The differences in sex assigned at birth and victimization levels found in the first step may explain why the data were not MCAR. Given the low missing rates, missing data were handled using FIML estimation, which performs with the least bias for Missing At Random and even Missing Not At Random data (Enders, 2022; Tang & Tong, 2024).

**References**

Alavi, M., Visentin, D. C., Thapa, D. K., Hunt, G. E., Watson, R., & Cleary, M. (2020). Chi-square for model fit in confirmatory factor analysis. *Journal of Advanced Nursing*, *76*(9), 2209–2211. https://doi.org/10.1111/jan.14399

Enders, C. K. (2022). *Applied Missing Data Analysis*. Guilford Publications.

Tang, D., & Tong, X. (2024). A Comparison of Full Information Maximum Likelihood and Machine Learning Missing Data Analytical Methods in Growth Curve Modeling. In M. Wiberg, J.-S. Kim, H. Hwang, H. Wu, & T. Sweet (Eds.), *Quantitative Psychology* (pp. 99–107). Springer Nature Switzerland. https://doi.org/10.1007/978-3-031-55548-0_10

Wheaton, B., Muthén, B., Alwin, D. F., & Summers, G. F. (1977). Assessing Reliability and Stability in Panel Models. *Sociological Methodology*, *8*, 84–136. https://doi.org/10.2307/270754

Step 1:

W7 full sample

*n_1_* = 10,952

Step 2:

W7 valid youth survey data

*n_2_* = 10,345

Step 3:

W7 Valid reports on SGD items

*n_3_* = 10,106

Step 4:

W4-W6 at least one valid parent-youth

or teacher-youth dyad

*n_4_* = 9936 (Analytical sample)

**Figure S1**

Participants selection flow diagram

| **Table S2** | | | | | | | |
| --- | --- | --- | --- | --- | --- | --- | --- |
| Number of valid victimization reports | | | | | | | |
| Model | Valid reports from | W4 | | W5 | | W6 | |
|  |  | n | valid % | n | valid % | n | valid % |
| Parent-youth | Both | 8260 | 89.76 | 8846 | 93.17 | 8755 | 94.38 |
|  | Only youth | 465 | 5.05 | 466 | 4.91 | 342 | 3.69 |
|  | Only parent | 477 | 5.18 | 182 | 1.92 | 179 | 1.93 |
| Teacher-youth | Both | 5708 | 63.39 | 5354 | 56.95 |  |  |
|  | Only youth | 3017 | 33.50 | 3958 | 42.10 |  |  |
|  | Only teacher | 280 | 3.11 | 89 | 0.95 |  |  |

| **Table S3** | | | | |
| --- | --- | --- | --- | --- |
| Mean level of victimization: Included and excluded samples in pairwise deletion | | | | |
| Wave | Youth-reported victimization | | Parent-reported victimization | |
|  | With parent-report | Without parent-report | With youth-report | Without youth-report |
| W4 | **1.56** | **1.71** | **1.22** | **1.3** |
| W5 | 2.32 | 2.25 | 1.28 | 1.36 |
| W6 | 2.02 | 1.94 | **1.27** | **1.52** |
| Wave | Youth-reported victimization | | Teacher-reported victimization | |
|  | With teacher-report | Without teacher-report | With youth-report | Without youth-report |
| W4 | 1.57 | 1.58 | 1.09 | 1.11 |
| W5 | **2.35** | **2.25** | **1.13** | **1.25** |
| *Note:* Bold indicates significant between-group differences in the independent sample T-test | | | | |

| **Table S4** | | | | |
| --- | --- | --- | --- | --- |
| Missing rate | | | | |
| Variable | Wave 4 | Wave 5 | Wave 6 | Wave 7 |
| Emotional problems | 11.8% | 6.1% | 8.4% | 0.1% |
| Self-esteem |  | 6.2% | 8.5% | 0.2% |

**Supplementary Appendix S5. Descriptive Analysis and Correlation**

| **Table S5** | | | | | | | | | | | | | | | | | | |
| --- | --- | --- | --- | --- | --- | --- | --- | --- | --- | --- | --- | --- | --- | --- | --- | --- | --- | --- |
| Descriptive Analysis and Correlation | | | | | | | | | | | | | | | | | | |
| Variables | | Range | Mean | SD | 1 | 2 | 3 | 4 | 5 | 6 | 7 | 8 | 9 | 10 | 11 | 12 | 13 | 14 |
| 1 | Y_vic (W4) | 1-3 | 1.57 | 0.64 | - |  |  |  |  |  |  |  |  |  |  |  |  |  |
| 2 | Y_vic (W5) | 1-6 | 2.31 | 1.58 | 0.18 ^***^ |  |  |  |  |  |  |  |  |  |  |  |  |  |
| 3 | Y_vic (W6) | 1-6 | 2.02 | 1.42 | 0.1 ^***^ | 0.32 ^***^ |  |  |  |  |  |  |  |  |  |  |  |  |
| 4 | P_vic (W4) | 1-3 | 1.22 | 0.48 | 0.24 ^***^ | 0.14 ^***^ | 0.1 ^***^ |  |  |  |  |  |  |  |  |  |  |  |
| 5 | P_vic (W5) | 1-3 | 1.28 | 0.54 | 0.17 ^***^ | 0.34 ^***^ | 0.18 ^***^ | 0.31 ^***^ |  |  |  |  |  |  |  |  |  |  |
| 6 | P_vic (W6) | 1-3 | 1.28 | 0.56 | 0.12 ^***^ | 0.22 ^***^ | 0.27 ^***^ | 0.23 ^***^ | 0.4 ^***^ |  |  |  |  |  |  |  |  |  |
| 7 | T_vic (W4) | 1-3 | 1.09 | 0.35 | 0.07 ^***^ | 0.07 ^***^ | 0.06 ^***^ | 0.14 ^***^ | 0.11 ^***^ | 0.1 ^***^ |  |  |  |  |  |  |  |  |
| 8 | T_vic (W5) | 1-3 | 1.13 | 0.40 | 0.09 ^***^ | 0.18 ^***^ | 0.09 ^***^ | 0.1 ^***^ | 0.26 ^***^ | 0.18 ^***^ | 0.08 ^***^ |  |  |  |  |  |  |  |
| 9 | Emo (W4) | 1-3 | 1.74 | 0.33 | 0.19 ^***^ | 0.08 ^***^ | 0.07 ^***^ | 0.06 ^***^ | 0.04 ^***^ | 0.01 | 0.01 | 0 |  |  |  |  |  |  |
| 10 | Emo (W5) | 1-5 | 1.96 | 0.67 | 0.14 ^***^ | 0.46 ^***^ | 0.23 ^***^ | 0.1 ^***^ | 0.21 ^***^ | 0.15 ^***^ | 0.06 ^***^ | 0.13 ^***^ | 0.12 ^***^ |  |  |  |  |  |
| 11 | Emo (W6) | 1-3 | 1.43 | 0.45 | 0.05 ^***^ | 0.17 ^***^ | 0.38 ^***^ | 0.05 ^***^ | 0.11 ^***^ | 0.17 ^***^ | 0.04 ^**^ | 0.03 ^*^ | 0.06 ^***^ | 0.25 ^***^ |  |  |  |  |
| 12 | Emo (W7) | 1-5 | 2.21 | 0.82 | 0.06 ^***^ | 0.15 ^***^ | 0.26 ^***^ | 0.06 ^***^ | 0.11 ^***^ | 0.12 ^***^ | 0.04 ^**^ | 0.04 ^**^ | 0.04 ^***^ | 0.2 ^***^ | 0.47 ^***^ |  |  |  |
| 13 | SE (W4) | 1-4 | 3.39 | 0.43 | -0.09 ^***^ | -0.23 ^***^ | -0.13 ^***^ | -0.08 ^***^ | -0.14 ^***^ | -0.13 ^***^ | -0.05 ^***^ | -0.05 ^***^ | -0.07 ^***^ | -0.35 ^***^ | -0.2 ^***^ | -0.15 ^***^ |  |  |
| 14 | SE (W5) | 1-4 | 3.12 | 0.58 | -0.04 ^***^ | -0.1 ^***^ | -0.24 ^***^ | -0.05 ^***^ | -0.08 ^***^ | -0.11 ^***^ | -0.02 | -0.01 | -0.05 ^***^ | -0.18 ^***^ | -0.6 ^***^ | -0.37 ^***^ | 0.3 ^***^ |  |
| 15 | SE (W6) | 1-4 | 3.00 | 0.64 | -0.05 ^***^ | -0.1 ^***^ | -0.16 ^***^ | -0.06 ^***^ | -0.07 ^***^ | -0.09 ^***^ | -0.03 ^*^ | -0.01 | -0.04 ^***^ | -0.13 ^***^ | -0.34 ^***^ | -0.54 ^***^ | 0.19 ^***^ | 0.39 ^***^ |
| *Note.* Pare-wise deletion was applied for correlation analysis. | | | | | | | | | | | | | | | | | | |
| Abbreviations: Y, youth-reported; P, parents-reported; T, teachers-reported; Vic, peer victimization; Emo, emotional problems; SE, self-esteem; W, wave. | | | | | | | | | | | | | | | | | | |
| ^*^ *p* < .05, ^**^ *p* < .01, ^***^ *p* < .001. | | | | | | | | | | | | | | | | | | |

**Supplementary Appendix S6. Post-hoc ANOVA**

| **Table S6** | | | | | |
| --- | --- | --- | --- | --- | --- |
| Post-hoc ANOVA: Mean differences for youth-reported peer victimization frequency | | | | | |
| Victimization class | Youth-reported frequency Mean (Standard Deviation) | | | | |
|  | Parent-youth model | | | Teacher-youth model | |
|  | Wave 4 | Wave 5 | Wave 6 | Wave 4 | Wave 5 |
| Congruent class | 2.25 (0.43) | 3.85 (1.57) | 3.61 (1.57) | 2.24 (0.43) | 3.92 (1.58) |
| Only-youth class | 2.14 (0.35) | 3 (1.32) | 2.81 (1.25) | 2.16 (0.37) | 3.17 (1.4) |
| Mean Difference | 0.11^***^ | 0.85^***^ | 0.80^***^ | 0.09^***^ | 0.75^***^ |
| ^***^ *p* < .001. | | | | | |

**Supplementary Appendix S7. Main and Carry-over Effects**

| **Table S7** | | | | | | | | | | |
| --- | --- | --- | --- | --- | --- | --- | --- | --- | --- | --- |
| Victim vs non-victim model with sexual orientation status: Main and carry-over effects | | | | | | | | | | |
| Paths | Emotional problems | | | | | | Self-esteem | | | |
|  | Wave 4 (Age 7) | | Wave 5 (Age 11) | | Wave 6 (Age 14) | | Wave 5 (Age 11) | | Wave 6 (Age 14) | |
|  | *β* | 95% CI | *β* | 95% CI | *β* | 95% CI | *β* | 95% CI | *β* | 95% CI |
| Model: Parent-youth reports | | | | | | | | | | |
| SDiv → MH (Wn) | 0.03^**^ | [0.01, 0.05] | 0.09^***^ | [0.05, 0.13] | 0.15^***^ | [0.12, 0.18] | -0.04^*^ | [-0.06, -0.01] | -0.13^***^ | [-0.17, -0.09] |
| Main → MH (Wn) | 0.04^***^ | [0.02, 0.06] | 0.08^***^ | [0.04, 0.11] | 0.12^***^ | [0.09, 0.15] | -0.02 | [-0.04, 0.01] | -0.11^***^ | [-0.14, -0.07] |
| SDiv → MH (Wn+1) | 0.18^***^ | [0.14, 0.23] | 0.18^***^ | [0.15, 0.21] | 0.39^***^ | [0.33, 0.44] | -0.16^***^ | [-0.2, -0.12] | -0.22^***^ | [-0.26, -0.17] |
| Main → MH (Wn+1) | 0.11^***^ | [0.07, 0.15] | 0.14^***^ | [0.11, 0.17] | 0.32^***^ | [0.27, 0.36] | -0.13^***^ | [-0.16, -0.1] | -0.12^***^ | [-0.16, -0.09] |
| Freq → MH (Wn) | 0.06^***^ | [0.03, 0.09] | 0.17^***^ | [0.15, 0.18] | 0.1^***^ | [0.09, 0.11] | -0.05^***^ | [-0.06, -0.04] | -0.07^***^ | [-0.08, -0.05] |
| Freq → MH (Wn+1) | 0.15^***^ | [0.09, 0.21] | 0.01^*^ | [0, 0.02] | 0.03^**^ | [0.01, 0.05] | 0 | [-0.02, 0.01] | -0.02^*^ | [-0.03, 0] |
| MH (Wn) → MH (Wn+1) | 0.2^***^ | [0.15, 0.24] | 0.14^***^ | [0.12, 0.15] | 0.65^***^ | [0.61, 0.69] | 0.36^***^ | [0.33, 0.39] | 0.38^***^ | [0.35, 0.4] |
| Model: Teacher-youth reports | | | | | | | | | | |
| SDiv → MH (Wn) | 0.03 | [0, 0.05] | 0.12^***^ | [0.06, 0.17] | |  | -0.06^**^ | [-0.1, -0.02] | |  |
| Main → MH (Wn) | 0.05^***^ | [0.02, 0.07] | 0.08^***^ | [0.04, 0.13] | |  | -0.02 | [-0.06, 0.01] | |  |
| SDiv → MH (Wn+1) | 0.2^***^ | [0.14, 0.26] | 0.2^***^ | [0.16, 0.25] | |  | -0.17^***^ | [-0.22, -0.11] | |  |
| Main → MH (Wn+1) | 0.1^***^ | [0.05, 0.15] | 0.14^***^ | [0.1, 0.18] | |  | -0.12^***^ | [-0.17, -0.08] | |  |
| Freq → MH (Wn) | 0.06^**^ | [0.03, 0.1] | 0.16^***^ | [0.15, 0.18] | |  | -0.05^***^ | [-0.06, -0.04] | |  |
| Freq → MH (Wn+1) | 0.13^**^ | [0.06, 0.21] | 0.02^**^ | [0.01, 0.03] | |  | -0.01 | [-0.02, 0.01] | |  |
| MH (Wn) → MH (Wn+1) | 0.23^***^ | [0.18, 0.29] | 0.13^***^ | [0.11, 0.15] | |  | 0.38^***^ | [0.34, 0.42] | |  |
| Abbreviations: *β*, standardized regression coefficients; CI, confidence interval; Sdiv, Sexually diverse; Main, Mainly heterosexual; Freq, youth-reported victimization frequency; MH, mental health outcomes. | | | | | | | | | | |
| ^*^ *p* < .05, ^**^ *p* < .01, ^***^ *p* < .001. | | | | | | | | | | |

| **Table S8** | | | | | | | | | | |
| --- | --- | --- | --- | --- | --- | --- | --- | --- | --- | --- |
| Victim vs non-victim model with gender identity status: Main and carry-over effects | | | | | | | | | | |
| Paths | Emotional problems | | | | | | Self-esteem | | | |
|  | Wave 4 (Age 7) | | Wave 5 (Age 11) | | Wave 6 (Age 14) | | Wave 5 (Age 11) | | Wave 6 (Age 14) | |
|  | *β* | 95% CI | *β* | 95% CI | *β* | 95% CI | *β* | 95% CI | *β* | 95% CI |
| Model: Victim vs non-victim model: Parent-youth reports with gender identity status | | | | | | | | | | |
| GD → MH (Wn) | 0.03 | [-0.04, 0.09] | 0.13^*^ | [0, 0.26] | 0.22^***^ | [0.13, 0.32] | 0.01 | [-0.08, 0.09] | -0.13^*^ | [-0.27, 0] |
| GD → MH (Wn+1) | 0.15^*^ | [0.01, 0.3] | 0.29^***^ | [0.19, 0.4] | 0.59^***^ | [0.44, 0.74] | -0.25^***^ | [-0.39, -0.11] | -0.41^***^ | [-0.54, -0.28] |
| Freq → MH (Wn) | 0.06^***^ | [0.03, 0.09] | 0.17^***^ | [0.15, 0.18] | 0.1^***^ | [0.09, 0.11] | -0.05^***^ | [-0.06, -0.04] | -0.07^***^ | [-0.08, -0.06] |
| Freq → MH (Wn+1) | 0.15^***^ | [0.08, 0.21] | 0.01^*^ | [0, 0.02] | 0.04^***^ | [0.02, 0.05] | -0.01 | [-0.02, 0.01] | -0.02^**^ | [-0.03, -0.01] |
| MH (Wn) → MH (Wn+1) | 0.21^***^ | [0.16, 0.25] | 0.14^***^ | [0.13, 0.16] | 0.7^***^ | [0.65, 0.74] | 0.36^***^ | [0.33, 0.39] | 0.39^***^ | [0.36, 0.41] |
| Abbreviations: *β*, standardized regression coefficients; CI, confidence interval; GD, gender diverse; Freq, youth-reported victimization frequency; MH, mental health outcomes. | | | | | | | | | | |
| ^*^ *p* < .05, ^**^ *p* < .01, ^***^ *p* < .001. | | | | | | | | | | |

| **Table S9** | | | | | | | | | | |
| --- | --- | --- | --- | --- | --- | --- | --- | --- | --- | --- |
| Single informant vs congruent model with sexual orientation status: Main and carry-over effects | | | | | | | | | | |
| Paths | Emotional problems | | | | | | Self-esteem | | | |
|  | Wave 4 (Age 7) | | Wave 5 (Age 11) | | Wave 6 (Age 14) | | Wave 5 (Age 11) | | Wave 6 (Age 14) | |
|  | *β* | 95% CI | *β* | 95% CI | *β* | 95% CI | *β* | 95% CI | *β* | 95% CI |
| Model: *Only-youth* vs *Congruent* (Parent and youth) | | | | | | | | | | |
| Freq → MH (Wn) | 0.06^***^ | [0.03, 0.09] | 0.17^***^ | [0.15, 0.18] | 0.1^***^ | [0.09, 0.11] | -0.05^***^ | [-0.06, -0.04] | -0.07^***^ | [-0.08, -0.05] |
| SDiv → MH (Wn) | 0.01 | [-0.02, 0.04] | 0.1^***^ | [0.05, 0.15] | 0.16^***^ | [0.12, 0.2] | -0.02 | [-0.06, 0.01] | -0.14^***^ | [-0.19, -0.08] |
| Main → MH (Wn) | 0.02 | [0, 0.05] | 0.08^**^ | [0.03, 0.12] | 0.12^***^ | [0.08, 0.15] | 0 | [-0.04, 0.03] | -0.09^***^ | [-0.13, -0.05] |
| Freq → MH (Wn+1) | 0.15^***^ | [0.09, 0.22] | 0.01^*^ | [0, 0.02] | 0.03^***^ | [0.02, 0.05] | -0.01 | [-0.02, 0.01] | -0.02^*^ | [-0.03, 0] |
| SDiv → MH (Wn+1) | 0.17^***^ | [0.1, 0.24] | 0.19^***^ | [0.15, 0.23] | 0.42^***^ | [0.36, 0.49] | -0.14^***^ | [-0.19, -0.09] | -0.24^***^ | [-0.29, -0.19] |
| Main → MH (Wn+1) | 0.14^***^ | [0.07, 0.2] | 0.14^***^ | [0.11, 0.18] | 0.28^***^ | [0.23, 0.34] | -0.13^***^ | [-0.17, -0.08] | -0.11^***^ | [-0.15, -0.06] |
| MH (Wn) → MH (Wn+1) | 0.22^***^ | [0.15, 0.3] | 0.13^***^ | [0.11, 0.15] | 0.62^***^ | [0.57, 0.67] | 0.34^***^ | [0.31, 0.38] | 0.4^***^ | [0.37, 0.44] |
| Model: *Only-parent* vs *Congruent* (Parent and youth) | | | | | | | | | | |
| Freq → MH (Wn) | 0.05 | [0, 0.09] | 0.18^***^ | [0.16, 0.2] | 0.1^***^ | [0.08, 0.12] | -0.06^***^ | [-0.07, -0.04] | -0.06^***^ | [-0.08, -0.04] |
| SDiv → MH (Wn) | 0.04 | [0, 0.09] | 0.13^**^ | [0.05, 0.21] | 0.16^***^ | [0.1, 0.22] | -0.06^*^ | [-0.12, -0.01] | -0.13^***^ | [-0.2, -0.06] |
| Main → MH (Wn) | 0.05^*^ | [0, 0.09] | 0.1^*^ | [0.01, 0.18] | 0.14^***^ | [0.08, 0.2] | -0.04 | [-0.1, 0.02] | -0.13^**^ | [-0.21, -0.06] |
| Freq → MH (Wn+1) | 0.15^**^ | [0.05, 0.26] | 0.03^**^ | [0.01, 0.04] | 0.03^*^ | [0, 0.06] | -0.01 | [-0.03, 0.01] | -0.02 | [-0.04, 0] |
| SDiv → MH (Wn+1) | 0.18^**^ | [0.08, 0.29] | 0.24^***^ | [0.18, 0.3] | 0.4^***^ | [0.31, 0.5] | -0.17^***^ | [-0.24, -0.09] | -0.23^***^ | [-0.3, -0.15] |
| Main → MH (Wn+1) | 0.09 | [-0.01, 0.2] | 0.2^***^ | [0.14, 0.26] | 0.26^***^ | [0.16, 0.36] | -0.14^***^ | [-0.22, -0.07] | -0.11^**^ | [-0.19, -0.04] |
| MH (Wn) → MH (Wn+1) | 0.2^**^ | [0.08, 0.31] | 0.11^***^ | [0.08, 0.14] | 0.67^***^ | [0.59, 0.76] | 0.28^***^ | [0.23, 0.34] | 0.36^***^ | [0.3, 0.42] |
| Model: *Only-youth* vs *Congruent* (Teacher and youth) | | | | | | | | | | |
| Freq → MH (Wn) | 0.06^**^ | [0.03, 0.1] | 0.17^***^ | [0.15, 0.18] | |  | -0.05^***^ | [-0.06, -0.04] | |  |
| SDiv → MH (Wn) | 0 | [-0.03, 0.04] | 0.12^**^ | [0.05, 0.18] | |  | -0.05 | [-0.09, 0] |  |  |
| Main → MH (Wn) | 0.05^**^ | [0.02, 0.08] | 0.08^**^ | [0.02, 0.14] | |  | -0.01 | [-0.05, 0.04] | |  |
| Freq → MH (Wn+1) | 0.22^***^ | [0.13, 0.31] | 0.02^**^ | [0.01, 0.03] | |  | -0.01 | [-0.02, 0.01] | |  |
| SDiv → MH (Wn+1) | 0.16^***^ | [0.07, 0.25] | 0.21^***^ | [0.15, 0.26] | |  | -0.15^***^ | [-0.22, -0.09] | |  |
| Main → MH (Wn+1) | 0.11^**^ | [0.04, 0.18] | 0.14^***^ | [0.09, 0.18] | |  | -0.11^***^ | [-0.17, -0.06] | |  |
| MH (Wn) → MH (Wn+1) | 0.14^***^ | [0.06, 0.21] | 0.12^***^ | [0.09, 0.15] | |  | 0.37^***^ | [0.32, 0.41] |  |  |
| Abbreviations: *β*, standardized regression coefficients; CI, confidence interval; Sdiv, Sexually diverse; Main, Mainly heterosexual; Freq, youth-reported victimization frequency; MH, mental health outcomes. | | | | | | | | | | |
| ^*^ *p* < .05, ^**^ *p* < .01, ^***^ *p* < .001. | | | | | | | | | | |

| **Table S10** | | | | | | | | | | |
| --- | --- | --- | --- | --- | --- | --- | --- | --- | --- | --- |
| Single informant vs congruent model with gender identity status: Main and carry-over effects | | | | | | | | | | |
| Paths | Emotional problems | | | | | | Self-esteem | | | |
|  | Wave 4 (Age 7) | | Wave 5 (Age 11) | | Wave 6 (Age 14) | | Wave 5 (Age 11) | | Wave 6 (Age 14) | |
|  | *β* | 95% CI | *β* | 95% CI | *β* | 95% CI | *β* | 95% CI | *β* | 95% CI |
| Model: *Only-youth* vs *Congruent* (Parent and youth) | | | | | | | | |  |  |
| Freq → MH (Wn) | 0.06^***^ | [0.03, 0.09] | 0.17^***^ | [0.15, 0.18] | 0.1^***^ | [0.09, 0.11] | -0.05^***^ | [-0.06, -0.04] | -0.07^***^ | [-0.08, -0.06] |
| GD → MH (Wn) | -0.02 | [-0.11, 0.06] | 0.15 | [-0.01, 0.31] | 0.26^***^ | [0.15, 0.37] | -0.04 | [-0.16, 0.07] | -0.14 | [-0.29, 0.02] |
| Freq → MH (Wn+1) | 0.15^***^ | [0.08, 0.21] | 0.01^**^ | [0, 0.02] | 0.04^***^ | [0.02, 0.06] | -0.01 | [-0.02, 0.01] | -0.02^**^ | [-0.03, -0.01] |
| GD → MH (Wn+1) | 0.16 | [-0.04, 0.36] | 0.32^***^ | [0.2, 0.45] | 0.52^***^ | [0.36, 0.68] | -0.23^**^ | [-0.4, -0.07] | -0.37^***^ | [-0.52, -0.22] |
| MH (Wn) → MH (Wn+1) | 0.23^***^ | [0.15, 0.3] | 0.14^***^ | [0.12, 0.16] | 0.66^***^ | [0.61, 0.71] | 0.35^***^ | [0.31, 0.39] | 0.41^***^ | [0.38, 0.45] |
| Abbreviations: *β*, standardized regression coefficients; CI, confidence interval; GD, gender diverse; Freq, youth-reported victimization frequency; MH, mental health outcomes. | | | | | | | | | | |
| ^*^ *p* < .05, ^**^ *p* < .01, ^***^ *p* < .001. | | | | | | | | | | |
